# Enhancing Performance and Bit Rates in a Brain–Computer Interface System With Phase-to-Amplitude Cross-Frequency Coupling: Evidences From Traditional c-VEP, Fast c-VEP, and SSVEP Designs

**DOI:** 10.3389/fninf.2018.00019

**Published:** 2018-05-08

**Authors:** Stavros I. Dimitriadis, Avraam D. Marimpis

**Affiliations:** ^1^Division of Psychological Medicine and Clinical Neurosciences, School of Medicine, Cardiff University, Cardiff, United Kingdom; ^2^Cardiff University Brain Research Imaging Centre, School of Psychology, Cardiff University, Cardiff, United Kingdom; ^3^School of Psychology, Cardiff University, Cardiff, United Kingdom; ^4^Neuroinformatics Group, Cardiff University Brain Research Imaging Centre, School of Psychology, Cardiff University, Cardiff, United Kingdom; ^5^Neuroscience and Mental Health Research Institute, Cardiff University, Cardiff, United Kingdom; ^6^Brain Innovation B. V., Maastricht, Netherlands

**Keywords:** brain–computer interface, c-VEP, SSVEP, disabled subjects, cross-frequency coupling, accuracy, phase-to-amplitude coupling, performance

## Abstract

A brain–computer interface (BCI) is a channel of communication that transforms brain activity into specific commands for manipulating a personal computer or other home or electrical devices. In other words, a BCI is an alternative way of interacting with the environment by using brain activity instead of muscles and nerves. For that reason, BCI systems are of high clinical value for targeted populations suffering from neurological disorders. In this paper, we present a new processing approach in three publicly available BCI data sets: (a) a well-known multi-class (*N* = 6) coded-modulated Visual Evoked potential (c-VEP)-based BCI system for able-bodied and disabled subjects; (b) a multi-class (*N* = 32) c-VEP with slow and fast stimulus representation; and (c) a steady-state Visual Evoked potential (SSVEP) multi-class (*N* = 5) flickering BCI system. Estimating cross-frequency coupling (CFC) and namely δ-θ [δ: (0.5–4 Hz), θ: (4–8 Hz)] phase-to-amplitude coupling (PAC) within sensor and across experimental time, we succeeded in achieving high classification accuracy and Information Transfer Rates (ITR) in the three data sets. Our approach outperformed the originally presented ITR on the three data sets. The bit rates obtained for both the disabled and able-bodied subjects reached the fastest reported level of **324 bits/min** with the PAC estimator. Additionally, our approach outperformed alternative signal features such as the relative power (29.73 bits/min) and raw time series analysis (24.93 bits/min) and also the original reported bit rates of **10–25 bits/min**. In the second data set, we succeeded in achieving an average ITR of 124.40 ± 11.68 for the slow 60 Hz and an average ITR of 233.99 ± 15.75 for the fast 120 Hz. In the third data set, we succeeded in achieving an average ITR of 106.44 ± 8.94. Current methodology outperforms any previous methodologies applied to each of the three free available BCI datasets.

## Introduction

From the very first work of Farwell and Donchin (Farwell and Donchin, 1988), the majority of P300-based brain–computer interface (BCI) systems focused on creating new applications (Polikoff et al., 1995; Bayliss, 2003), and on constructing and testing new algorithms for the reliable detection of the P300 waveform from noisy data sets (Xu et al., 2003; Kaper et al., 2004; Rakotomamonjy et al., 2005; Thulasidas et al., 2006; Hoffmann et al., 2008). For a review of P300, an interested person can read the following: Reza et al. (2012), Farwell et al. (2013), and Piccione et al. (2006).

The majority of BCI systems are based on three major types of brain signals: the event-related resynchronization which is associated with the P300, the motor-imagery and the steady-state visual evoked potentials (SSVEP) (Wolpaw et al., 2002). P300 could give very good results, for example in a spelling device (Guger et al., 2009), but for continuous control in a daily scenario like steering a wheelchair in many different directions, SSVPE performed better (Lin and Kuo, 2016).

Other approaches to the traditional BCI systems are based on visual evoked potentials (VEPs) paradigms. VEPs are alternative brain signals that can be used in a BCI system. Frequently, the two methods are mostly employed to distinguish various visual targets, the phase and frequency coding (Wang et al., 2008). These VEPs are usually observed in the occipital area in response to a repetitive visual stimulus, and they encode the undergoing visual information processing in the brain. In the context of BCIs, a subject is focused (fixated) into a flashing image (target). Each target is coded differently and thus, is presented by a unique stimulus sequence. These results are unique visual responses easily identified in the brain activity. BCI VEP-based systems can be organized into three distinct categories depending on the design: time (t), frequency (f), and (c) code modulated. The reader is invited to consult review works such as Riechmann et al. (2016) for more details. In this work, we will focus on a c-VEP system in which pseudorandom sequences are used for presenting the stimuli.

The most common domain where BCI c-VEPs are employed is the matrix spellers. It has been previously shown that they outperform the traditional BCIs regarding ITR performance (Mohebbi et al., 2015; Riechmann et al., 2016) with classification accuracies also being comparable.

c-VEPs are just starting to gain popularity in another domain; that is the control of virtual or physical devices. In Mohebbi et al. (2015), the authors built a system in which a 12-target virtual agent was simulated in a 3D environment (accuracy around 80%). Their paradigm closely follows those using the classic matrix design, but they replaced the letters with navigation and interaction symbols accordingly. A real-world scenario is developed in Kapeller et al. (2013a) where users can control (in real time) a remote robot with reported accuracies up to 98.18%; though only four navigational symbols (left, right, forward, backward) were used. In the same spirit, an application was developed to control a wheelchair model in four directions (Mohebbi et al., 2015). Their subject-specific study reported an average of 97% accuracy when controlling the wheelchair.

A novel c-VEP study has proposed a high presentation rate of coding sequence up to 120 Hz compared to the traditional 60 Hz. This is a very significant study since the interface was based on 32 circular white targets following a sequence stimulation paradigm. Apart from the frequency of the coding sequence, they also introduced a novel decoding algorithm based on spatio-temporal beamforming. Wittevrongel et al. (2017) reported that the median ITR was 172.87 bits/min.

The core of BCI-SSVEP systems is based on oscillatory responses elicited when a light source flashes at a specific frequency (e.g., 60 Hz). These frequency-dependent responses are spatially oriented over the parieto-occipital cortex. The design of a BCI-SSVEP system is multi-targeted where each one flashes on a different frequency (Müller-Putz et al., 2005) or on the same frequency (Maye et al., 2017). The subject has to focus on one of the targets that are presented simultaneously. The outcome of the SSVEP response is the translation of the subject's decision tailored to the design of the BCI system like a speller (Chen et al., 2015; Maye et al., 2017).

The bibliography suggests that the dominant methodology in c-VEP studies (Kapeller et al., 2013a,b; Mohebbi et al., 2015; Riechmann et al., 2016) is the usage of spatial filters through Canonical Correlation Analysis (CCA) combined with a classification algorithm (most notably SVMs Farwell and Donchin, 1988; Spuller et al., 2012a or LDA Polikoff et al., 1995). Briefly, CCA finds projections of the original EEG signal to increase the distinct activity among EEG sensors and it is used as a common spatial filter (Kapeller et al., 2013a). A recent retinotopic multi-target SSVEP study adopted CCA as spatial filters of amplitude and phase domain of the single trials, achieving very high classification accuracy (Maye et al., 2017).

Thus, researchers are experimenting with modifying the protocol to achieve optimal results. For instance, in Bin et al. (2009), they experimented with different EEG buffer lengths (in seconds) to produce the CCA templates. The authors reported an improved accuracy score up to 99.21%; however, this configuration has a direct impact on the latency of the BCI system. Another more sophisticated approach is explored in (Spuller et al., 2012b), where the authors incorporated the Error-related Potentials to initially calibrate the system online; thus, directing the classifier to the correct class. This approach achieved a grand average accuracy of 96.18%.

Hoffmann et al. demonstrated a six-choice P300 paradigm which was tested in a group of five disabled and four able-bodied subjects. The experimental paradigm was six flashing images with the content of a home device (Hoffmann et al., 2008). They tested how the electrode configuration can influence the accuracy in order to detect the best channel selection. They finally succeeded in achieving increased communication rates and classification accuracies compared to previous studies (Piccione et al., 2006; Sellers and Donchin, 2006; McCane et al., 2015).^1^

Multiple feature extraction techniques have been used in BCI systems including the analysis of raw time series, the estimation of signal power, connectivity analysis and so on. The most famous algorithms include the fast fourier transform (Resalat and Saba, 2016), the Auto-Regressive Model (Pineda et al., 2003), the short-time fourier transform, and the wavelet decomposition (Nguyen et al., 2015). Compared to the past, brain connectivity attracts much attention for BCI systems (Kabbara et al., 2016). However, cross-frequency coupling (CFC) has not yet explored its potentiality to BCI systems and especially to c-VEP and SSVEP BCI systems.

In the present study, we used the data set from Hoffmann et al. to demonstrate an alternative algorithmic approach with the main scope of improving the bit rates up to the limits. Our study focused on the c-VEP subcomponent of the brain signals generated by the flashing images. The basic hypothesis is to decode the features from the brain activity that are directly related to the content of the flashing image. For that occasion, we adopted a CFC estimator, namely phase-to-amplitude coupling (PAC), to quantify how the phase of the lower frequency brain rhythms modulates the amplitude of the higher oscillations. The whole approach was followed on a trial basis and within sensors located over the parieto-occipital brain areas. PAC proved to be a valuable estimator in many applications like the design of a biomarker: for amnestic mild cognitive impairment subjects during an auditory oddball paradigm (Dimitriadis et al., 2015), for dyslexia (Dimitriadis et al., 2016) and for mild traumatic brain injury (Antonakakis et al., 2016). Our main goal is to improve the performance and the bit rates focusing on the c-VEP component of the brain activity.

To further enhance the proposed methodology based on CFC-PAC estimates, we also report the results from two freely available BCI data sets. The first data set is a c-VEP multi-target (32 targets) gaze BCI system with slow (60 Hz) and fast (120 Hz) stimulus representation (Wittevrongel et al., 2017). The second data set is a SSVEP and is associated with flickering stimuli at different frequencies (5 frequencies−5 targets) with the main scope of predicting the gaze direction (Georgiadis et al., 2018).

To the best of our knowledge, this work is the only one suggesting CFC features for a BCI system and especially for c-VEP and SSVEP.

The layout of the paper is as follows. In section Materials and Methods, we briefly describe the three EEG data sets, the subject population, the experimental set-up, the methods used for data preprocessing steps of the proposed pipeline and the classification procedure. The results are presented in section Results. The discussion is addressed in section Discussion.

## Materials and methods

### c-VEP flashing images data set

#### Experimental set-up

**Figure 1:**
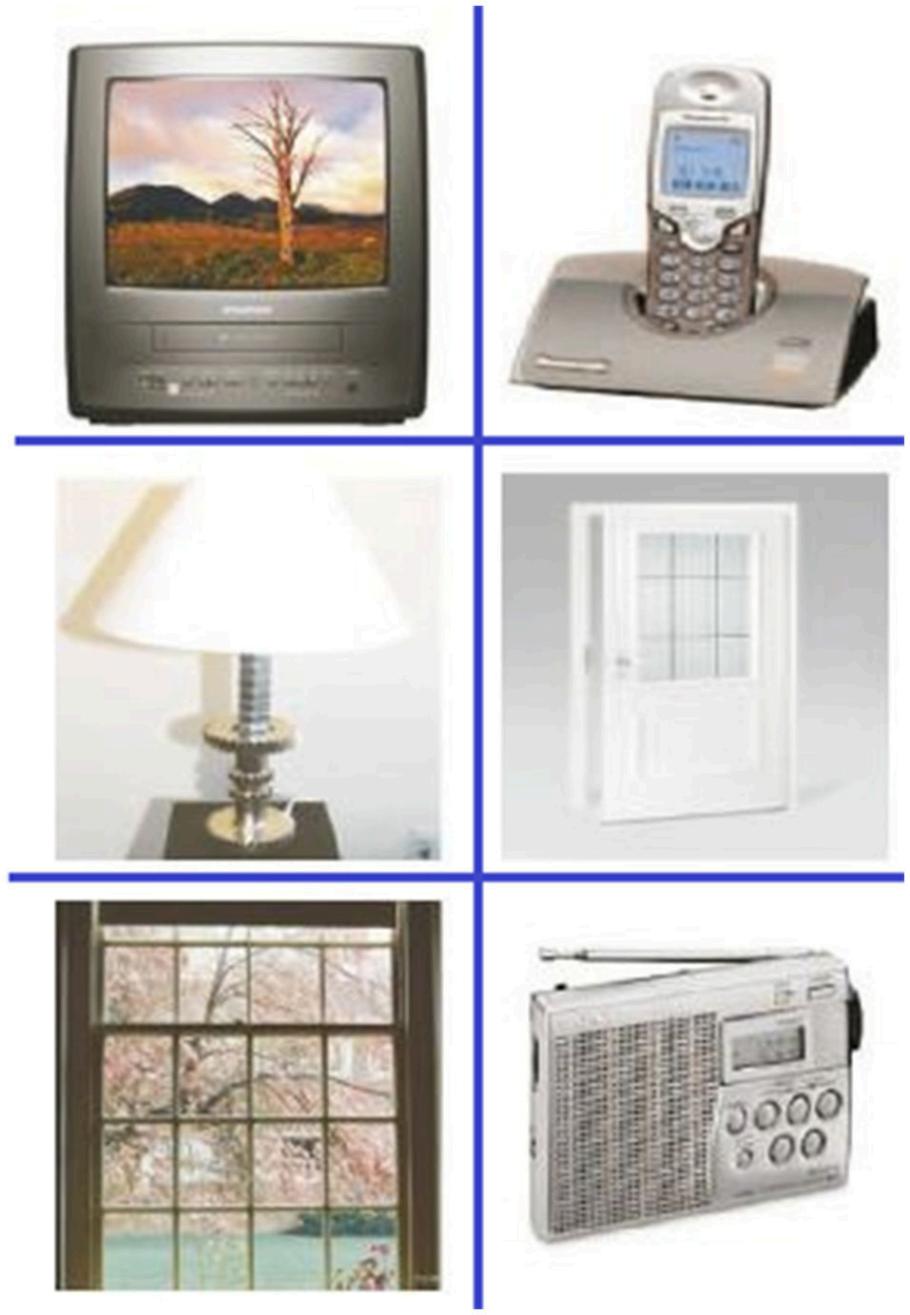
The six flashing images.

Six targeted flashed images are illustrated in Figure 1. The images show: a television, a telephone, a lamp, a door, a window, and a radio. The images were flashed for 100 ms and during the following 300 ms, none of the images was flashed, i.e., the inter-stimulus-interval was 400 ms.

The EEG was recorded at a 2048 Hz sampling rate from 32 EEG sensors placed at the standard positions of the 10–20 international system. For further details see the original paper (Hoffmann et al., 2008).

#### Subjects

The system was tested with five disabled and four healthy subjects. The disabled subjects were all wheelchair-bound but had varying communication and limb muscle control abilities (see Table 1). Subjects 1–4 are the disabled group where in Table 1 one can see their description. Subjects 6–9 were Ph.D. students recruited from EPFL BCI group's laboratory (all males, age 30 ± 2.3 years). None of subjects 6–9 had known neurological deficits. Communication with Subject 5 was very difficult due to a severe hypophony and large fluctuations in the level of alertness.

**Table 1:** Subjects from which data were recorded in the study of the environment control system (Hoffmann et al., 2008).

	**S1**	**S2**	**S3**	**S4**
Diagnosis	Cerebral palsy	Multiple sclerosis	Late-stage amyotrophic lateral sclerosis	Traumatic brain and spinal-cord injury, C4 level
Age	56	51	47	33
Age at illness onset	0 (perinatal)	37	39	27
Sex	M	M	M	F
Speech production	Mild dysarthria	Mild dysarthria	Severe dysarthria	Mild dysarthria
Limb muscle control	Weak	Weak	Very Weak	Weak
Respiration control	Normal	Normal	Weak	Normal
Voluntary eye movement	Normal	Mild nystagmus	Normal	Normal

#### Experimental schedule

Each subject recruited to participate in this study completed four recording sessions, two sessions on 1 day and the remaining two sessions on a second day, with a maximum of 2 weeks between the two recording days. Each recording session consisted of a total number of six runs, one run per targeted image (Figure 1).

The duration of each run was 1 min and of the recording session was around 30 min. One session included on average 810 trials, while the whole data for each subject consisted of, on average, 3,240 trials. For further details about the protocol see Hoffmann et al. (2008).

#### Preprocessing of single trials

The impact of different single-sensor recordings on classification accuracy was tested in an offline procedure.

Before learning a classification function and cross-validation scheme, several preprocessing operations were applied to the data.

The preprocessing steps applied to the data set in this study are presented in the following steps:

- *Referencing*. We re-referenced single trials using the average signal from the two mastoid electrodes.
- *Filtering*. A third-order zero phase Butterworth bandpass filter was used to filter the data. The MATLAB function *butter* was used to compute the filter coefficients and the function *filtfilt* was used for filtering. The predefined frequencies were: δ {0.5–4 Hz}, θ {4–8 Hz}, α_1_ {8–10 Hz}, α_2_ {10–13 Hz}, β_1_ {13–20 Hz}, β_2_ {20–30 Hz}, and γ_1_ {30–45 Hz}.
- *Downsampling*. The EEG was downsampled from 2,048 to 512 Hz.
- *Single trial extraction*. Single trials have a duration of 1,000 ms from the stimulus onset up to 1,000 ms after the stimulus onset.
- *Electrode selection*. We applied our analysis to recordings from single-sensor activity and mainly, PZ, OZ, P3, P4, P7, and P8.
- *Feature vector construction*. As an appropriate feature for each trial, we used PAC which has already shown its potentiality in building reliable biomarkers (Dimitriadis et al., 2015, 2016). PAC was estimated for each frequency pair (see ii). The description of PAC is given in the next section. As a complementary feature that can separate the counted stimuli from the non-counted stimuli, α relative signal powers have been estimated. Alpha power level can give us a valuable and objective criterion when a subject attends or does not attend the stimulus. Our idea is to create an initial binary classifier that will cut-off the attended from the non-attended stimuli for each subject prior to entering the main multi-class classifier.

##### CFC metric computation

CFC quantifies the strength of interactions between a time series of different frequency content. It can be estimated both within and also between sensors (Buzsáki, 2010; Canolty and Knight, 2010; Buzsáki et al., 2013). CFC can be estimated between power—power, amplitude—amplitude, and amplitude-phase representations of two time series with different frequency content. These representations can be derived by filtering twice one (within) or once two time series (between). The most common type of CFC interaction is PAC and it is the most common in the literature (Voytek et al., 2010). The PAC algorithm for a single EEG sensor is described below.

Let x(i_sensor_, t), be the EEG time series at the i_sensor_-th recording site, and *t* = 1, 2,.…T the sample points. Given a bandpassed filtered signal x(i_sensor_,t), CFC is quantified under the notion that the phase of the lower frequency (LF) oscillations modulate the amplitude of the higher frequency (HF) oscillations. The following equations described the complex representations of both LF z_LF_(t) and HF oscillations z_HF_(t) produced via the Hilbert transform (HT):

$$\begin{array}{l} {\text{z}}_{\text{LF}}(\text{t})=\text{HT}\left[{\text{x}}_{\text{LF}}(\text{t})\right]=|{\text{z}}_{\text{LF}}(\text{t})|\;{\text{e}}^{\text{i}\varphi_{\text{LF}}\left(t\right)}=A_{\text{LF}}(\text{t}){\text{e}}^{\text{i}\varphi_{\text{LF}}\left(t\right)},\\ \;{\text{z}}_{\text{HF}}(\text{t})=\text{HT}\left[{\text{x}}_{\text{HF}}(\text{t})\right]=|{\text{z}}_{\text{HF}}(\text{t})|\;{\text{e}}^{\text{i}\varphi_{\text{HF}}\left(t\right)}=A_{\text{HF}}(\text{t}){\text{e}}^{\text{i}\varphi_{\text{HF}}\left(t\right)} \end{array}$$

The next step of the PAC algorithm is the estimation of the envelope of the HF oscillation A_HF_(t) which is then bandpass-filtered within the frequency range of LF oscillations. Afterward, the resulting time series is again Hilbert transformed in order to get its phase time series that describe phase dynamics ϕ'(t):

$$\begin{array}{l} z^{\prime }\left(t\right)=HT\left[A_{HF,LF}\left(t\right)\right]=|z^{\prime }\left(t\right)|e^{i\varphi_{HF}^{\prime }\left(t\right)}\\ =|z^{\prime }\left(t\right)|e^{i\varphi_{LF\to HF}\left(t\right)} \end{array}$$

The aforementioned complex equation describes analytically the modulation of the amplitude of HF oscillation by the phase of LF oscillation.

The phase consistency between those two time series can be measured by the original phase-locking value (PLV) estimator (Lachaux et al., 1999) but also from its imaginary portion of PLV. The imaginary part of PLV (iPLV) can be used as an synchronization index that quantifies the strength of CFC-PAC coupling.

PLV is defined as follows:

(1) $$\begin{array}{l} PLV=\frac{1}{T}*\overset{T}{\underset{t=1}{\sum }}e^{t\left(\phi_{LF}\left(t\right)-\phi_{HF}\left(t\right)\right)} \end{array}$$

and the iPLV as follows:

(2) ImPLV=1T*|Im(∑t=1Tei(ϕLF(t)-ϕHF(t)))|

The iPLV is an estimator that is less affected compared to PLV from the volume conduction effect. Using iPLV for quantifying the strength of CFC interactions is an advantage over volume conduction. The iPLV is more sensitive to non-zero phase lag and for that reason is more resistant to any self-interactions that are directly linked to volume conductions (Nolte et al., 2004). For further details and applications, an interested reader can read our previous work Dimitriadis et al. (2015, 2016).

**Figure 2:**
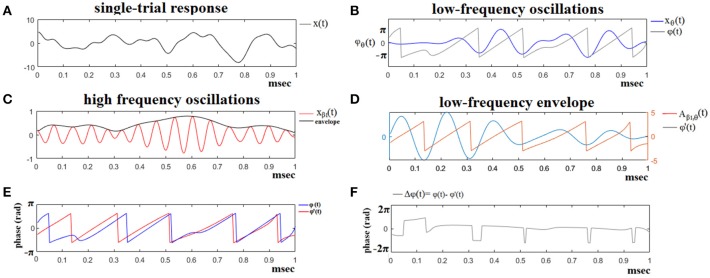
The algorithmic steps for PAC estimation. Using the first single-trial signal from session 1 and flashing image 1 **(A)**, from the P300 of an able subject (subject 6), we demonstrate the detection of coupling between θ and β1 rhythm. To estimate θ-β1 PAC, the raw signal was band-pass filtered into both a **(B)** low-frequency θ (4–8 Hz) component where its envelope is extracted as well as **(C)** a high-frequency β1 (13–20 Hz) component where its instantaneous phase is extracted. **(D)** We then extracted the amplitude and the instantaneous phase of the band-passed β1 (13–20 Hz) and filtered this amplitude time series at the same frequency as θ (4–8 Hz), giving us the θ modulation in lower β amplitude. **(E)** We then extracted the instantaneous phase of both the θ-filtered signal and the θ-filtered lower-β amplitude and computed the phase-locking between these two signals. The latency depended differences **(F)**, will be used in estimating the phase-locking (iPLV) that will reflect the PAC-interaction between the two involved brain rhythms. This phase-locking represents the degree to which the lower β (β1) amplitude is co-modulated with the θ phase.

In the present study, as already mentioned, we used seven frequency bands which means that PAC is estimated for 7^*^6/2 = 21 cross-frequency pairs e.g., δ^ϕ^-θ^A^, δ^ϕ^-$\alpha_{1}^{\text{A}}$ where ϕ and A denote the phase and amplitude of each frequency band. Figure 2 demonstrates the preprocessing steps of the PAC estimator for a trial of Subject 6 at Target Image 6.

For comparison purposes, we estimated the CFC phase-to-amplitude estimates via two alternative approaches: (1) In the first one, we followed the same analytic pathway as the one described above but instead of the imaginary part of PLV, PLV was estimated and (2) in the second approach, Canolty's et al. (2006) definitions were adopted based on mean vector length (MVL) and the complex estimation of modulation of the phase of slower rhythm to the amplitude of the higher oscillation. Hereafter, we will use the terms of PAC^iPLV^, PAC^PLV^ and PAC^MVL^ to describe the CFC-PAC-based estimates with the three approaches.

We estimated the three different CFC estimates (PAC^iPLV^, PAC^PLV^ and PAC^MVL^) and relative signal power (RSP) for the first 32 samples (60 ms) increasing the window up to 500 ms (256 samples) with a step of 12 samples (5 ms).

##### Signal power

We estimated the relative power of each bandpass frequency signal segment with the following equations:

(3) $$\begin{array}{l} SP\left(fr\right)=\overset{T}{\underset{t=1}{\sum }}filtered{\left(t\right)}^{2} \end{array}$$

(4) $$\begin{array}{l} RSP\left(fr\right)=\frac{SP\left(fr\right)}{\sum_{fr=1}^{frequencies}SP\left(fr\right)} \end{array}$$

The first equation quantifies the signal power (SP) of each frequency as the sum of the filtered signal squared per sample (Equation 3) while Equation (4) divides the SP by the sum of the SP from all the frequencies which gives the RSP. The whole approach was repeated for every trial, sessions and subject. For the RSP estimation, we used the same predefined frequencies as for CFC-PAC estimates.

##### Machine learning and classification

The training data set includes on average 405/2025 target/non-target trials and the validation data sets consisted of 135/675 target/non-target trials.

Adopting, we used an unsupervised multi-class feature selection algorithm (Cai et al., 2010) to detect the characteristic cross-frequency pair via PAC value that gives the highest discrimination of each target image compared to the rest based on the training data set. Additionally, we used a sequential feature selection algorithm to detect the RSP that separate the counted flashing images from the non-counted images.

We trained a multi-class SVM classifier based on the selected PAC estimate from specific cross-frequency pairs and then we tested the classifier to the validation data to get the response tailored to each target image (Joachims, 1999). The training test consisted of the first session while the remaining three sessions were used for validating the whole analytic scheme. A k-nearest neighbor (k-NN) classifier was applied to differentiate the attended from the non-attended flashing images prior to a multi-class SVM classifier based on α RSP.

### c-VEP slow/fast stimulus presentation

#### Experimental set-up

The time course of one trial of the experiment can be seen in Figure 1 in Wittevrongel et al. (2017). The design of the interface consisted of 32 circular white targets following an m-sequence stimulation paradigm (see further) and that were overlaid with static (i.e., non-flickering) gray letters or numbers arranged in a matrix of 4 (row) × 8 (columns).

The following m-sequence of a length of 63 was used to encode the targets:

$$\begin{array}{l} 000100001011001010100100111100.00011011100110001110\\ 1011111101101 \end{array}$$

where targets were lagged by integer multiples of two frames.

A trial started with the presentation of a target cue. Subjects were instructed to redirect their gaze to the cued target and then to press a button to start the trial/stimulation. After that, all targets were hidden but the characters were still shown in gray for 1 s, followed by the stimulation phase during which all targets adopted their unique lagged m-sequence and repeated this sequence either five or ten times for slow and fast stimulus representation, respectively.

The EEG was recorded at a 250 Hz sampling rate from 32 EEG sensors placed at the standard positions of the 10–20 international system. For further details see the original paper (see Figure 2 in Wittevrongel et al., 2017).

#### Subjects

Seventeen subjects with normal or corrected-to-normal vision participated in the experiment (14 female, 13 right handed, aged 22.35 ± 2.9, ranging from 18 to 30 years old). The data set and the preprocessing steps followed on from the original papers which are publicly available.^2^

#### Experimental schedule

Every subject performed 5/10 m-sequence repetitions per trial for a 60/120 Hz stimulation rate. The total duration of a trial was 5.25 s.

The original goal of this study was dual. First, to assess the performance of the spatio-temporal beamforming algorithm for target identification when using cVEP-based encoding, and secondly to compare the performance for both slow-traditional (60 Hz) and high-speed (120 Hz) stimulus presentations (Wittevrongel et al., 2017).

#### Preprocessing of single trials

The impact of different single-sensor recordings on classification accuracy was tested in an offline procedure.

Before learning a classification function and cross-validation scheme, several preprocessing operations were applied to the data.

The preprocessing steps applied to the data set in this study are presented in the following steps:

- *Referencing*. We re-referenced single trials using the average reference signal instead of using the average signal from the mastoid as in the original data set.
- *Filtering*. A third-order zero phase Butterworth bandpass filter was used to filter the data. The MATLAB function *butter* was used to compute the filter coefficients and the function *filtfilt* was used for filtering. The predefined frequencies were: δ {0.5–4 Hz}, θ {4–8 Hz}, α_1_ {8–10 Hz}, α_2_ {10–13 Hz}, β_1_ {13–20 Hz}, β_2_ {20–30 Hz}, and γ_1_ {30–45 Hz}.
- *Single trial extraction*. Single trials have a duration of 5250 ms (5.25 s) from the stimulus onset up.
- *Electrode selection*. We applied our analysis to recordings from single-sensor activity using the whole set of 32 EEG recording channels.
- *Beamforming*. We adopted the same strategy as in the original paper by building beamformers based on the training epochs for each subject. Beamformers act as spatial filterers and have shown their potentiality in event-related potential (ERP) studies (van Vliet et al., 2016). The activation patterns and the target and frequency specific beamformers were calculated from the training data $T_{training}\in R^{m\times t\times t}$ where m is the number of channels, t is the number of samples and l is the number of epochs, as follows. For each epoch in training, a maximal number of t-second consecutive non-overlapping segments were extracted, where t represents the time needed to display one complete m-sequence.
  The whole procedure was followed independently for each subject, target and frequency.
  An LCMV beamformer was finally estimated for each target and frequency based on the testing data set. In the original paper, they applied the proposed beamformer within 4–31 Hz. For further details, see the original paper.
  Our goal was not to use beamformers as a classifier but to diminish the effect of spurious activity among the EEG sensor channels.
- *Feature vector construction*. As an appropriate feature for each trial, we used PAC which already has shown its potentiality in building reliable biomarkers (Dimitriadis et al., 2015, 2016). PAC was estimated for each frequency pair (see ii). We used a sliding-window of 100 ms (25 samples) that moved every 0.004 s (one sample). This approach leads to a PAC time series (PAC^ts^) of 501 samples long. Finally, for every subject and for each target, we have estimated a matrix with the following dimensions: trials x sensors (32) × CFC-pairs (21) x PAC^ts^ (501). Trials refer to the testing data set following a five-fold cross-validation procedure.

Preprocessing steps have been applied independently to slow and fast stimulus representation.

#### Machine learning and classification

For each subject and at every five-fold of the cross-validation procedure, we thoroughly searched for the optimal set of channel selection, CFC-pairs and the time needed (length of PAC^ts^) to reach a plateau of classification performance or 100% absolute accuracy. Apart from the classification performance, time is an important parameter that further increases the optimal information transfer rate (ITR).

At every fold, we encoded the single trial PAC^ts^ in the training set via a symbolisation procedure based on the neural-gas algorithm (Martinetz et al., 1993). This approach has already been used in single trial responses in a BCI-SSVEP system (Georgiadis et al., 2018) and also for transforming dynamic functional brain networks into functional connectivity microstates (Dimitriadis et al., 2013). Practically, for each PAC^ts^ and for each sensor, we designed encoded prototypical code waves for the training data set.

To access the recognition accuracy of each channel and each PAC^ts^ across the CFC-pairs and across time t (samples of the PAC^ts^), we employed the **Wald-Wolfowitz (WW) test** as a similarity index between training prototypical PAC^ts^ and PAC^ts^ from every single trial of the testing set. For further details regarding the WW-test, see section 2 in the Supplementary Material. The time window across the PAC^ts^ was moved per sample in order to detect the best classification accuracy in a shorter time.

### SSVEP multi-target data set

#### Experimental set-up

The visual stimulation included five violet squares, located as a cross (Figure 6 in Georgiadis et al., 2018) and flickering simultaneously at five different frequencies (6.66, 7.50, 8.57, 10.00, and 12.00 Hz).

The brain activity was recorded using a high-density EEG scanner with 256 electrodes [an EGI 300 Geodesic EEG System (GES 300)]. The sampling frequency was 250 Hz and the impedance for all electrodes was kept below 10 KΩ. During the recordings, an online bandpass filter (0.1 Hz−70 Hz) was applied to suppress noise and a 50 Hz notch filter to eliminate the power line interference (Georgiadis et al., 2018).

#### Subjects

Eleven healthy volunteers (8 males and 3 females mean ± SD age = 30.36 ± 5.20 years) participated in this study. The data set is publicly available.^3^

#### Experimental schedule

Each subject participated in five sessions with 25 flickering windows per session leading to 125 trials of 5 s in total and 25 trials per target frequency. The order of the flickering targets (gaze directions) was randomly chosen.

The original goal of this study was to access the recognition accuracy of the SSVEP BCI system using multi-targets flickering at different frequencies (Wittevrongel et al., 2017). The selection of the frequencies focused on avoiding frequencies that are multiples of another frequency.

#### Preprocessing of single trials

The impact of different single-sensor recordings on classification accuracy was tested in an offline procedure.

Before learning a classification function and cross-validation scheme, several preprocessing operations were applied to the data.

The preprocessing steps applied to the data set in this study are presented in the following steps:

- *Referencing*. We re-referenced single trials using the average reference signal.
- *Filtering*. A third-order zero phase Butterworth bandpass filter was used to filter the data. The MATLAB function *butter* was used to compute the filter coefficients and the function *filtfilt* was used for filtering. The predefined frequencies were: δ {0.5–4 Hz}, θ {4–8 Hz}, α_1_ {8–10 Hz}, α_2_ {10–13 Hz}, β_1_ {13–20 Hz}, β_2_ {20–30 Hz} and γ_1_ {30–45 Hz}.
- *Single trial extraction*. Single trials have a duration of 5000 ms (5 s) from the stimulus onset up.
- *Electrode selection*. We applied our analysis to recordings from single-sensor activity using the whole set of 52 parieto-occipital EEG recording channels (see Figure 6 in Oikonomou et al., 2016).
- *Beamforming*. We adopted the same strategy as in the second data set using the beamformers. Beamformers act as spatial filterers and have shown their potentiality in ERP studies (van Vliet et al., 2016). The activation patterns and the target and frequency specific beamformers were calculated from the training data$T_{training}\in R^{m\times t\times t}$, where m is the number of channels, t is the number of samples and l is the number of epochs, as follows. For each epoch in training, a maximal number of t-second consecutive non-overlapping segments were extracted, where t represents the time needed to display one complete m-sequence.
  The whole procedure was followed independently for each subject, target and frequency.
  An LCMV beamformer was finally estimated for each target and frequency based on the testing data set. For further details, see the original paper.
  Our goal was not to use beamformers as a classifier but to diminish the effect of spurious activity among the EEG sensor channels.
- *Feature vector construction*. As an appropriate feature for each trial, we used PAC, which has already shown its potentiality in building reliable biomarkers (Dimitriadis et al., 2015, 2016). PAC was estimated for each frequency pair (see ii). We used a sliding-window of 100 ms (25 samples) that moved every 0.004 s (one sample). This approach leads to a PAC time series (PAC^ts^) of 501 samples long. Finally, for every subject and for each target, we have estimated a matrix with the following dimensions: trials × sensors (25) × CFC-pairs (21) × PAC^ts^ (501). Trials refer to the testing data set following a five-fold cross-validation procedure.

Preprocessing steps have been applied independently to slow and fast stimulus representation.

#### Machine learning and classification

For each subject and at every five-fold of the cross-validation procedure, we thoroughly searched for the optimal set of channel selection, CFC-pairs and the time needed (length of PAC^ts^) to reach a plateau of classification performance or 100% absolute accuracy. Apart from the classification performance, time is an important parameter that further increases the optimal ITR.

At every fold, we encoded the single trial PAC^ts^ in the training set via a symbolisation procedure based on the neural-gas algorithm (Martinetz et al., 1993). This approach has already been used in single trial responses in a BCI-SSVEP system (Georgiadis et al., 2018) and also for transforming dynamic functional brain networks into functional connectivity microstates (Dimitriadis et al., 2013). Practically, for each PAC^ts^ and for each sensor, we designed encoded prototypical code waves for the training data set.

To access the recognition accuracy of each channel and each PAC^ts^ across the CFC-pairs and across time t (samples of the PAC^ts^), we employed the **WW-test** as a similarity index between training prototypical PAC^ts^ and PAC^ts^ from every single trial of the testing set. For further details regarding the WW-test, see section 2 in the Supplementary Material. The time window across the PAC^ts^ was moved per sample in order to detect the best classification accuracy in a shorter time.

### Performance evaluation

Classification accuracy and ITR were estimated for the offline experiments. We estimated ITR (in bits per second) with the following Equation (5):

(5) $$\begin{array}{l} B=\left(log2\left(N\right)+P*log2\left(P\right)+\left(1-P\right)*log2\left(\left(1-P\right)/\left(N-1\right)\right)\right)\\ ITR=B/\left(T/60\right) \end{array}$$

Where N is the number of classes/target images (i.e., six in this study), P is the accuracy of identification of the targeted image and t (seconds per selection) is the average time for a selection. ITR expressed the bits/symbol divided by the average time required to select a single symbol, T.

For data set 1, T = 0.4 s (300 ms duration of the flashing image + optimal time window from the response due to the decision) and *N* = 6 for both disabled and able-bodied subjects.

For data set 2, *N* = 32 and T was set to the optimal length of PAC^ts^ plus an additional 500 ms to account for the time the subject would need to switch their gaze to the next target.

For data set 3, *N* = 5 and T was set to the optimal length of PAC^ts^ plus an additional 5 s to account for the time the subject would need to switch their gaze to the next target.

## Results

### c-VEP flashing images data set

#### δ-θ PAC as a valuable feature for the BCI—c-VEP system

**Figure 3:**
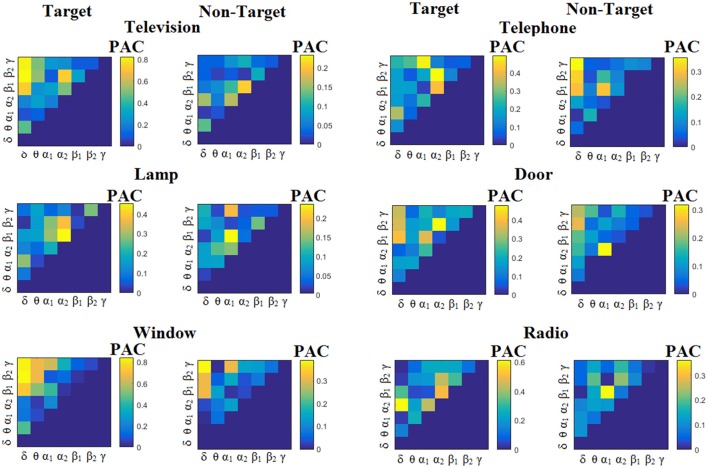
Subject 6 (able bodied). Demonstrating the level of CFC in c-VEP responses for each flashing image. Trial-Averaged **PAC**^iPLV^ patterns from the **c-VEP** responses for each target image and for both attended vs. non-attended images.

**Figure 4:**
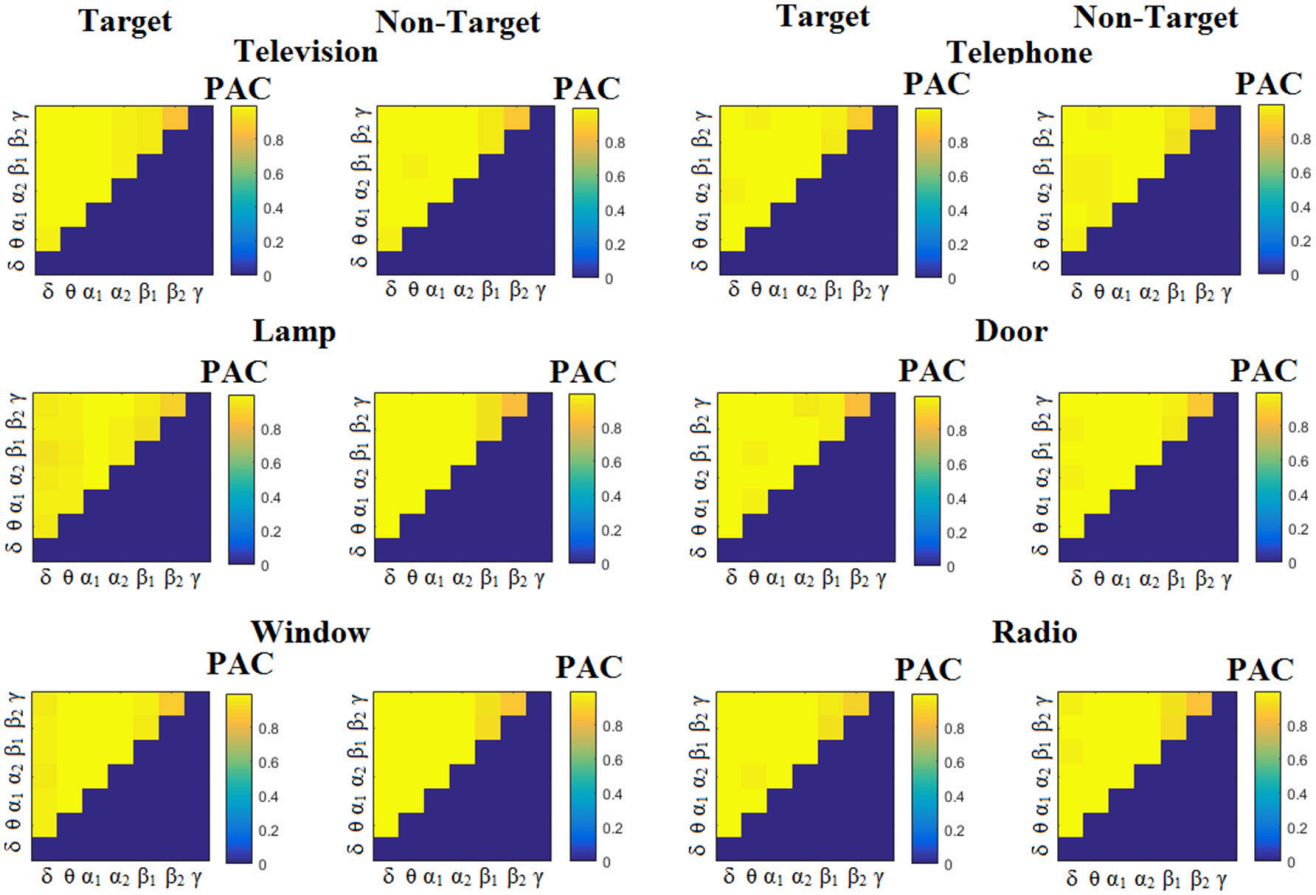
Subject 6 (able bodied). Demonstrating the level of CFC in c-VEP responses for each flashing image. Trial-Averaged **PAC**^PLV^ patterns from the **c-VEP** responses for each target image and for both attended vs. non-attended images.

**Figure 5:**
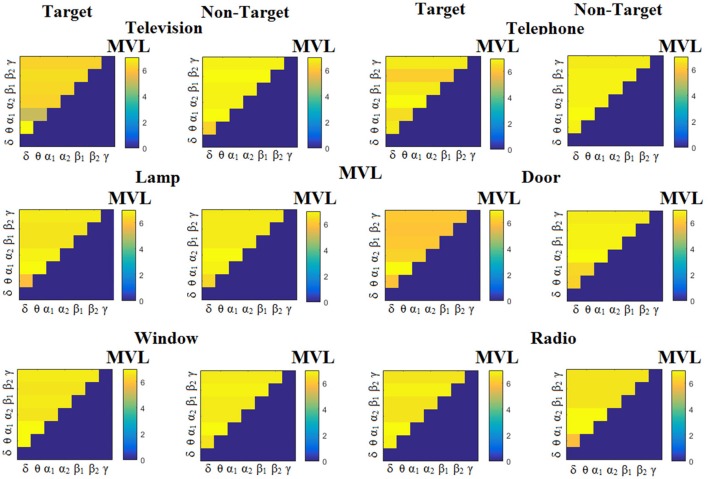
Subject 6 (able bodied). Demonstrating the level of CFC in c-VEP responses for each flashing image. Trial-Averaged **PAC**^MVL^ patterns from the **c-VEP** responses for each target image and for both attended vs. non-attended images.

**Figure 6:**
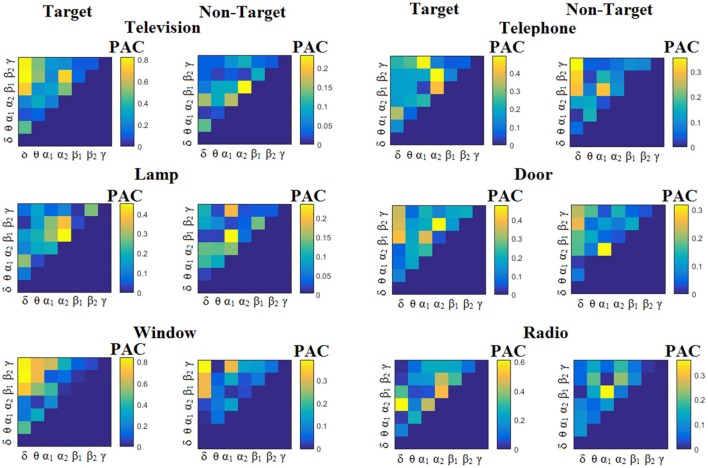
Subject 1(disabled). Demonstrating the level of CFC in c-VEP responses for each flashing image. Trial-Averaged **PAC**^iPLV^ patterns from the c-VEP responses for each target image and for both attended vs. non-attended images.

**Figure 7:**
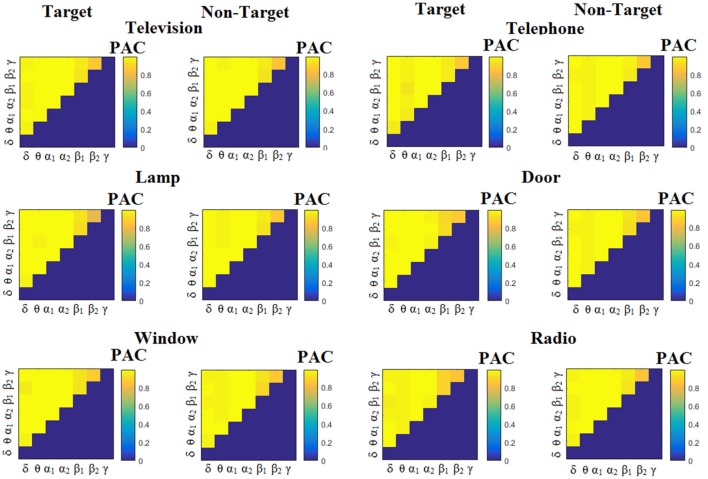
Subject 1(disabled). Demonstrating the level of CFC in c-VEP responses for each flashing image. Trial-Averaged **PAC**^PLV^ patterns from the **c-VEP** responses for each target image and for both attended vs. non-attended images.

**Figure 8:**
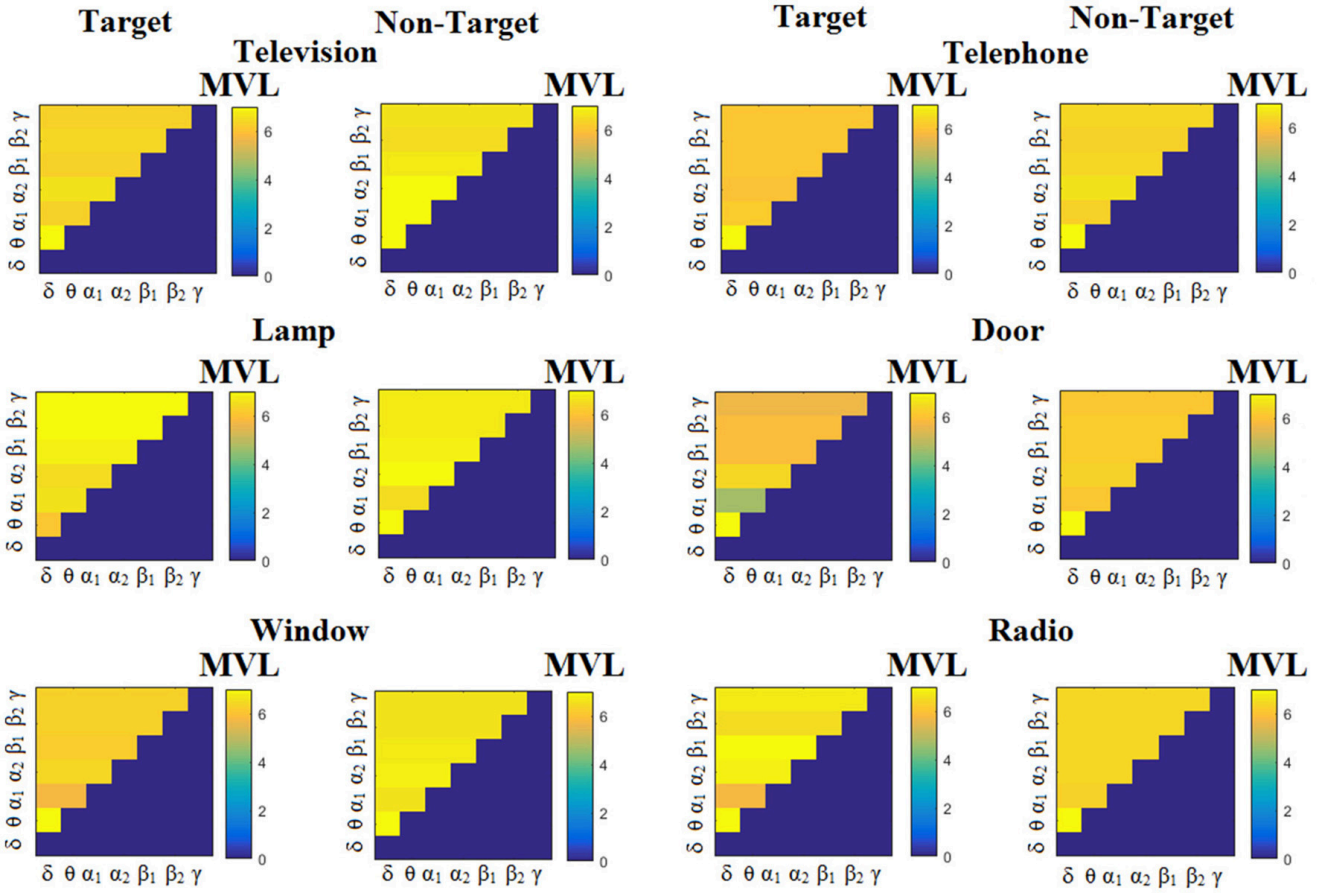
Subject 1(disabled). Demonstrating the level of CFC in c-VEP responses for each flashing image. Trial-Averaged **PAC**^MVL^ patterns from the **c-VEP** responses for each target image and for both attended vs. non-attended images.

We estimated both PAC and RSP for the first 32 samples (60 ms) increasing the window up to 500 ms (256 samples) with a step of 12 samples (5 ms). The multi-class unsupervised algorithm (Cai et al., 2010) detected only one PAC feature from the 21 possible cross-frequency pairs as the unique candidate feature to separate the six classes of image stimuli. δ^ϕ^-θ^A^ was the selected feature for both disabled and able-bodied subjects for PAC^iPLV^. In contrast, the selected CFC features based on PAC^PLV^ and PAC^MVL^ were completely random and different between and also within subjects. Trial-averaged comodulograms of PAC values for the three adapted estimators are demonstrated for two our of eight subjects (see Figures 3–**5**, **6**–**8**). For the rest of the subjects see Supplementary Material.

The group-averaged classification performance was 99.96%±0.03 for each sensor location using the first 100 ms for both able-bodied and disabled subjects. The errors were detected on the trials where the subject missed the flashing image. The classification performance with the use of a kNN-classifier prior to the multi-class SVM was ~100 % for every subject and for all the pre-selected sensors namely PZ, OZ, P3, P4, P7, P8 EEG sensors.

Table 2 summarizes the classification accuracy for every subject and connectivity estimator with the related ITR based on the Pz EEG sensor. The ITR obtained for both the disabled and able-bodied subjects reached the fastest reported level of **6.45 bits/s (or 387 bits/min)** with the PAC^iPLV^ estimator compared to **3.79 bits/s (or 227 bits/min)** with the PAC^PLV^ estimator and **3.80 bits/s (or 228 bits/min)** with the PAC^MVL^ estimator. Additionally, our approach outperformed alternative signal features such as the RSP **(1.09–1.37 bits/s)** and raw time series analysis **(1.05–1.25 bits/s)** and also the original reported bit rates of **10–25 bits/min**. The new preprocessing approach was based on recordings from the single-sensor Pz, while the classification accuracy was also tested for at the other electrodes.

**Table 2:** PAC-CFC: Single-subject classification and the related bit rates for the disabled (Subjects 1–4) and able-bodied (Subjects 5–8) subjects based on the Pz sensor and the three alternative CFC-PAC estimators.

	**Classification accuracy (PAC^iPLV^/PAC^PLV^/PAC^MVL^)**	**ITR (PAC^iPLV^/PAC^PLV^/PAC^MVL^)**
Subject 1	99.91/83.03/82.44	385.87/230.08/226.03
Subject 2	99.92/82.35/82.55	386.05/225.417/226.78
Subject 3	99.96/82.35/82.45	386.84 /225.41/226.09
Subject 4	99.95/82.11/82.41	386.63/223.78/225.82
Subject 5	99.97/82.63/82.42	387.04/227.33/225.89
Subject 6	99.99/82.39/83.88	387.48/225.69/236.02
Subject 7	99.99/83.30/82.90	387.48/231.96/229.18
Subject 8	99.99/83.45/82.85	387.48/233.00/228.84

We detected a significant difference between ITR^iPLV^ and ITR original presented in Hoffmann et al. (2008) (Wilcoxon rank-sum test, *p* < 0.00001. Comparing ITR^iPLV^ with ITR^PLV^ and ITR^MVL^, we also detected significant differences (Wilcoxon rank-sum test, *p* < 0.00001). These results support the proposed iPLV estimator over the rest two.

Figure 3 illustrates the trial-related (grand-averaged) PAC^iPLV^ -connectivity patterns (comodulograms) from the first able-bodied subject from target and non-target trials for each flashing image. In contrast, Figures 4, 5 demonstrate, similarly to Figure 3, the grand-averaged PAC^PLV^-connectivity patterns and the grand-averaged PAC^MVL^-connectivity patterns, respectively (Canolty's et al., 2006).

Similarly, Figure 6 demonstrates the grand-averaged PAC-connectivity patterns of from the first disabled subject using the PAC^iPLV^ comodulograms. For comparison purposes, Figures 7, 8 are dedicated to the grand-averaged PAC^PLV^-connectivity patterns and the grand-averaged PAC^MVL^ -connectivity patterns, respectively (Canolty's et al., 2006). S.1–6 illustrate the grand-averaged PAC-connectivity patterns for the remaining six subjects of the data set (see Supplementary Material).

Comodulograms differed by contrasting target vs. non-target within each subject and target image but also between the two images. δ^ϕ^ −θ^A^ was the unique and consistent feature for both disabled and able-bodied subjects based on the PAC^iPLV^ that can clearly predict the target image for both groups. Comodulograms derived from PAC^PLV^ and PAC^MVL^ are more random without succeeding to differentiate the 21 cross-frequency pairs in both conditions and across the flashing images. This observation further supports the non-consistency of feature selection across individuals for those two alternative PAC-CFC estimators.

Applying a Wilcoxon Rank Sum test of trial-based δ^ϕ^ −θ^A^ between the able-bodied and disabled subjects, we detected significant different values (*p* < 0.01). Group-averaged δ^ϕ^ −θ^A^ were higher for able-bodied subjects compared to disabled in the six-targeted images. However, the dataset is too small in order to make any conclusion regarding the sensitivity of δ^ϕ^ −θ^A^ to detect abnormal visual decoding activity.

#### Attention and alpha power

Prior to multi-class SVM, we applied a kNN-classifier based on α_1_ SP which was selected as the feature that can discriminate counted from non-counted flashing images. The kNN-classifier performed 100% clear filtration of attended from non-attended trials for each subject and further improved the performance of multi-class SVM to 100%. We achieved this performance using an α_1_ signal relative power estimated from the first 100 ms for both able-bodied and disabled subjects.

The classification performance with the kNN-classifier was ~100% for every subject and for all the pre-selected sensors namely PZ, OZ, P3, P4, P7, P8 EEG sensors.

Table 3 summarizes the group-averaged RSP of an α_1_ frequency band for attended vs .non-attended images.

**Table 3:** Group-averaged α_1_ signal relative power for attended and non-attended images.

	**Attended**	**Non-attended**
Able-bodied	0.09 ± 0.02	0.06 ± 0.01
Disabled	0.10 ± 0.02	0.07 ± 0.01

#### Managing the cross-session transfer learning problem

In order to explore the effect on classification performance of collecting the data on two different days, we performed the same analysis using the trials derived from the second set of two sessions as a training set and the trials of the first set of two sessions as a testing set. **Table 6**, in complete analogy with Table 4, summarizes the classification accuracy and the ITR for every subject and CFC-PAC-connectivity estimator. The classification accuracy and bit rates diminished for the three CFC-PAC estimators compared to the original validation procedure (three sessions as a training set and the fourth as a testing set). Bit rates were: **5.4 bits/s for** PAC^iPLV^ (Mean Classification Accuracy: 94.63), **3.02 bits/s for** PAC^PLV^ (Mean Classification Accuracy: 75.40) and **3.03 bits/s for** PAC^MVL^ (Mean Classification Accuracy: 75.52). Classification performance was still higher for PAC^iPLV^ compared to the two alternatives, while classification performance was still higher than the two alternative estimators and too high to support our approach as a key feature in the c-VEP BCI system.

**Table 4:** PAC-CFC: Single-subject classification and the related bit rates for the disabled (Subjects 1–4) and able-bodied (Subjects 5–8) subjects based on the Pz sensor and the three alternative CFC-PAC estimators.

	**Classification accuracy (PAC^iPLV^/PAC^PLV^/PAC^MVL^)**	**ITR (min) (PAC^iPLV^/PAC^PLV^/PAC^MVL^)**
Subject 1	94.63/83.03/75.45	323.75/ 230.08/181.62
Subject 2	95.35/82.35/75.35	330.84/225.41 /181.03
Subject 3	95.15/82.35/75.15	328.85/225.41/179.86
Subject 4	96.62/82.11/75.61	344.00/223.78/182.57
Subject 5	94.12/82.63/75.32	318.86/227.33/180.86
Subject 6	92.51/82.39/76.09	304.06/225.69/185.43
Subject 7	93.20/83.30/75.80	310.29/231.96/183.70
Subject 8	95.45/83.45/75.44	331.85/233.00/181.57

δ^ϕ^ −θ^A^ was again the selected feature for both disabled and able-bodied subjects for PAC^iPLV^. In contrast, the selected CFC features based on PAC^PLV^ and PAC^MVL^ were completely random where in only two out of eight subjects, the selected feature was δ^ϕ^ −θ^A^.

We detected a significant difference between ITR^iPLV^ and ITR original presented in Hoffmann et al. (2008) (Wilcoxon rank-sum test, *p* < 0.00001. Comparing ITR^iPLV^ with ITR^PLV^ and ITR^MVL^, we also detected significant differences (Wilcoxon rank-sum test, *p* < 0.00001). These results support the proposed iPLV estimator over the rest two.

#### PAC-CFC vs. relative power—raw time series

To demonstrate the superiority of PAC-CFC to capture the local multiplexity of the human brain activity linked to c-VEP, we analyzed α_1_ relative power and raw time series filtered in α_1_. For comparison reasons, we used the first 100 ms after the end of the flashing images as we did with PAC-CFC. For the classification performance, we adopted multi-class Support Vector machines for both α_1_ relative power and α_1_ raw time series in order that the results be comparable with those derived from the three PAC-CFC estimators. Tables 5, 6 demonstrate the classification performance and ITR for each subject using recordings from the Pz sensor. Group-averaged bit rates for α_1_ relative power were 0.48 bits/s (29.73 bits/min) while for α_1_ raw time series were 0.41 bits/s (24.93 bits/min). Both alternative features extracted from the EEG recordings supported bit rates 12 times lower compared to the PAC^iPLV^ (**5.4 bits/s or 324 bits/min)**. In general, PAC-CFC outperformed both α_1_ relative power and α_1_ raw time series in improving the bit rates further.

**Table 5:** α_1_ Relative Power: Single-subject classification and the related bit rates for the disabled (Subjects 1–4) and able-bodied (Subjects 5–8) subjects based on the Pz sensor.

	**Classification accuracy**	**ITR (min)**
Subject 1	33.45	18.03
Subject 2	38.45	29.19
Subject 3	36.47	24.49
Subject 4	35.58	22.50
Subject 5	40.12	33.44
Subject 6	41.23	36.40
Subject 7	42.02	38.57
Subject 8	40.78	35.18

**Table 6:** α_1_ Raw time series: Single-subject classification and the related bit rates for the disabled (Subjects 1–4) and able-bodied (Subjects 5–8) subjects based on the Pz sensor.

	**Classification accuracy**	**ITR (min)**
Subject 1	32.12	15.4734
Subject 2	33.37	17.8800
Subject 3	35.51	22.3516
Subject 4	34.69	20.5863
Subject 5	38.34	28.9280
Subject 6	39.21	31.0982
Subject 7	39.87	32.7903
Subject 8	38.91	30.3421

We detected a significant difference between ITR^iPLV^ (Table 2) and α signal power (Table 5) and α raw time series (Table 6; Wilcoxon rank-sum test, *p* < 0.00001).

#### Performance evaluation

In the present study, we succeeded ITR of 324.06 bits/min [see Table 4; with *N* = 6, *P* = 94.63, and *T* = 0.4 s (300 ms duration of the flashing image + 100 ms time window from the response due to the stimulus)] for both disabled and able-bodied subjects correspondingly for the Pz sensor location. The time for estimation of PAC and testing the trial was 0.00001 s on a Windows 7 Intel 7–8-core machine.

### c-VEP slow/fast stimulus presentation

#### δ-θ PAC as a valuable feature for the BCI—c-VEP system

The group-averaged classification performance was 95.96 ± 2.15 for the 60 Hz and 95.91 ± 0.61 for the 120 Hz.

Table 7 summarizes the classification accuracy for every subject, the related ITR, the number of selected EEG sensors and the type of CFC-pairs. We succeeded an average ITR of 124.40 ± 11.68 for the slow 60 Hz and an average ITR of 233.99 ± 15.75 for the fast 120 Hz.

**Table 7:** PAC-CFC: Single-subject classification and the related bit rates for the 17 subjects based on the c-VEP data set in both slow and fast stimulus representation.

	**Classification accuracy (60/120 Hz)**	**ITR (min) (60/120 Hz)**
Subject 1	99.15 ± 1.61/96.31 ± 1.56	136.38 ± 4.31/247.13 ± 6.45
Subject 2	95.67 ± 1.87/95.78 ± 1.43	128.15 ± 4.53/227.63 ± 5.61
Subject 3	99.14 ± 1.11/94.67 ± 1.21	136.99 ± 3.99/253.50 ± 5.12
Subject 4	98.75 ± 1.23/95.01 ± 1.31	133.85 ± 3.69/225.98 ± 5.43
Subject 5	96.54 ± 1.87/95.45 ± 1.47	129.90 ± 3.44/235.97 ± 5.42
Subject 6	95.12 ± 1.66/96.07 ± 1.66	120.45 ± 4.11/230.65 ± 5.37
Subject 7	96.41 ± 1.37/96.12 ± 1.40	132.02 ± 3.91/242.76 ± 5.61
Subject 8	94.37 ± 1.48/95.88 ± 1.33	112.55 ± 3.77/212.20 ± 6.01
Subject 9	93.14 ± 1.39/95.76 ± 1.41	97.34 ± 3.81/227.67 ± 5.14
Subject 10	94.51 ± 1.18/96.62 ± 1.37	108.27 ± 3.97/211.47 ± 5.62
Subject 11	93.27 ± 1.31/96.34 ± 1.45	111.96 ± 4.01/213.90 ± 5.09
Subject 12	92.18 ± 1.48/97.01 ± 1.43	122.22 ± 4.31/236.45 ± 6.11
Subject 13	97.81 ± 1.34/96.38 ± 1.20	134.13 ± 3.92/242.03 ± 6.34
Subject 14	96.54 ± 1.41/95.43 ± 1.57	114.81 ± 3.87/222.05 ± 6.71
Subject 15	97.78 ± 1.45/95.17 ± 1.67	128.58 ± 3.67/225.47 ± 6.89
Subject 16	94.19 ± 1.32/96.07 ± 1.46	131.11 ± 3.77/260.92 ± 5.91
Subject 17	96.67 ± 1.24/96.51 ± 1.57	136.01 ± 4.11/262.00 ± 6.72

**Figure 9:**
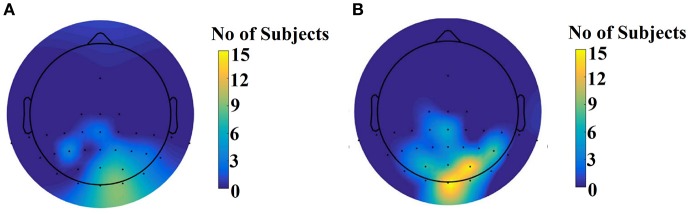
Scalp plot illustrating how many times each channel contributed to the best performance across subjects. **(A)** For the slow stimulation representation (60 Hz) and **(B)** For the fast stimulation representation (120 Hz).

Figure 9 illustrates the number of times each channel was selected across subjects for the slow stimulation representation (60 Hz) and for the fast stimulation representation (120 Hz).

We detected a significant difference between ITR^iPLV^ and ITR original presented in Wittevrongel et al. (2017) (Wilcoxon rank-sum test, *p* < 0.00001).

### SSVEP multi-target data set

#### δ-θ PAC as a valuable feature for the BCI—c-VEP system

The group-averaged classification performance was 94.25 ± 0.01 for the five targets.

Table 8 summarizes the classification accuracy for every subject, the related ITR, the number of selected EEG sensors and the type of CFC-pairs. We succeeded an average ITR of 106.44 ± 8.94.

**Table 8:** PAC-CFC: Single-subject classification and the related bit rates for the 11 subjects based on the SSVEP Multi-Target Data set.

	**Classification accuracy**	**ITR (min)**
Subject 1	99.15 ± 1.45	128.86 ± 5.61
Subject 2	95.67 ± 1.31	103.10 ± 3.43
Subject 3	99.14 ± 1.12	101.41 ± 3.12
Subject 4	98.75 ± 1.44	109.39 ± 3.97
Subject 5	96.54 ± 1.61	102.56 ± 4.01
Subject 6	95.12 ± 1.56	97.38 ± 3.46
Subject 7	96.41 ± 1.47	103.47 ± 3.91
Subject 8	94.37 ± 1.78	113.79 ± 4.82
Subject 9	93.14 ± 1.85	98.67 ± 3.79
Subject 10	94.51 ± 1.45	110.04 ± 4.11
Subject 11	93.27 ± 1.32	102.13 ± 4.78

**Figure 10:**
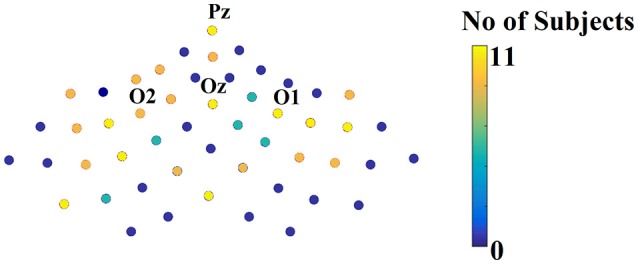
Scalp plot illustrating how many times each channel contributed to the best performance across subjects.

Figure 10 illustrates the number of times each channel was selected across subjects over the parieto-occipital brain areas.

We detected a significant difference between ITR^iPLV^ and ITR original presented in Georgiadis et al. (2018) (Wilcoxon rank-sum test, *p* < 0.00001).

## Discussion

A novel approach of how to analyse single trials in a BCI system was introduced based on the estimation of CFC and namely PAC. PAC was estimated within EEG sensors from single trials recorded during a visually evoked experimental paradigm. The proposed analytic scheme is based on the extraction of unique features from the CFC patterns on a single trial basis, namely the δ^ϕ^ −θ^A^ coupling. To evaluate the proposed analytic scheme and to further support the adaptation of CFC-PAC in BCI systems, we analyzed and presented our findings in three free publicly available EEG BCI data sets.

The first study referred to a well-known multi-class (*N* = 6) c-VEP-based BCI system for able-bodied and disabled subjects. Our experimentations showed a high classification rate (94.63%) based on the proposed PAC^iPLV^ estimator. In contrast, the two alternative PAC-CFC estimators succeeded in high classification accuracy and bit rates, but the choice of CFC features was random while the comodulograms were uniform across the cross-frequency pairs for every subject and flashing image.

The bit rates obtained for both the disabled and able-bodied subjects reached the fastest reported level of an ITR of **324 bits/min** with the PAC^δ−θ^ estimator. Additionally, our approach outperformed alternative signal features such as the relative power ITR = 9.73 bits/min and raw time series analysis ITR = **24.93 bits/min** and also the originally reported ITR = **10–25 bits/min**. Our results outperformed the results presented on the original (**324 bits/min vs 10–25 bits/min**; Hoffmann et al., 2008). Using a binary classifier trained with α_1_ RSP prior to the multi-class SVM, we differentiated the attended from the non-attended stimuli which further improved the classification performance up to 100% in both groups.

The success of PAC^iPLV^ was further enhanced by the poorer classification accuracy and bit rates of the two comparable approaches (PAC^PLV^, PAC^MVL^). This further supported our analytic signal processing for the estimation of PAC-CFC estimates and the use of iPLV instead of PLV and also compared to MVL. In our previous studies, using the PAC^iPLV^, we built a single-sensor and multi-sensor biomarker for mild cognitive impairment and reading disabilities using electro and magneto-encephalography recordings, respectively (Dimitriadis et al., 2015, 2016).

To properly manipulate any cross-session transfer between the two recording sessions, we repeated the whole classification analysis using, as a training set, the recordings from the second session and as a testing set, the recordings from the first session. The bit rates and the overall classification accuracy was decreased but the bit rates derived from the PAC^iPLV^ (5.4 bits/s) were still higher than the remaining two alternative CFC-PAC estimates and were kept at a very high level compared to other BCI studies. Overall, CFC-PAC estimates outperformed SP and raw time series analysis in α_1_ **frequency** further supporting the proposed analytic scheme.

In previous studies, like that of Sellers and Donchin (2006), the highest succeeded classification accuracy for the able-bodied and ALS subjects was 85 and 72%, respectively. Hoffmann et al. succeeded in absolute classification accuracy for both disabled and able-bodied subjects for the first demonstration of the current data set. However, they used a longer time series of over 15–20 s by concatenating trials in order to better train the classifier. Additionally, they used one classifier per image per each of the 20 blocks and the final outcome derived as the majority voting of the 20 classifiers.

The second BCI data set was a multi-class (*N* = 32) c-VEP with slow and fast stimulus representation. We succeeded in an average **ITR** = **124.40** ± **11.68 bits/min** for the slow 60 Hz and an average **ITR** = **233.99** ± **15.75 bits/min** for the fast 120 Hz. The major feature that contributes to this high classification accuracy was the PAC^δ−θ^. Our results outperformed the ITR presented in the original paper (Wittevrongel et al., 2017), while our results further supported the introduction of LCMV beamformers in the BCI system (Wittevrongel and Van Hulle, 2016a; Wittevrongel et al., 2017).

The third BCI data set was a SSVEP multi-class (*N* = 5) flickering BCI system where we succeeded in an average **ITR** = **106.44** ± **8.94 bits/min**. Like in the previous two data sets, the major feature that contributes to this high classification accuracy was the PAC^δ−θ^. Our results outperformed the ITR presented in the original paper (Georgiadis et al., 2018), where they analyzed five out of 11 subjects based on broadband activity after first encoding single trials via a symbolization approach. Their analysis focused on the classification performance using only one EEG sensor at a time and the highest accuracies were achieved from sensors located over the parieto-occipital brain area.

The core of the bibliography in c-VEP studies, Kapeller et al. (2013a,b), Mohebbi et al. (2015) and Riechmann et al. (2016) suggests that as a dominant methodology, the usage of spatial filters through CCA combined with a classification algorithm [most notably SVMs (Farwell and Donchin, 1988; Spuller et al., 2012a) or LDA Polikoff et al., 1995] is considered. Here, alternatively, we proposed the usage of CFC-PAC as a descriptor that quantifies the local multiplexity of brain functions as each one oscillates on a characteristic frequency. In a recent retinotopic multi-class with single flickering frequency, they proposed a CCA spatial filter of the EEG responses in both the amplitude and phase domain (Maye et al., 2017). They achieved a high classification accuracy even in the nine classes referring to different visual angles across a visual circle. Additionally, in recent years, many researchers have introduced the notion of beamformers as spatial filters of scalp EEG activity (Wittevrongel and Van Hulle, 2016b; Wittevrongel et al., 2017). The results presented are comparable and even superior to SVM. In the second and third data set, we estimated PAC time series after first applying a LCMV beamformer.

The majority of BCI studies analyzed broadband signal while they preferred to analyse the preprocessed broadband raw time series using first a symbolization scheme Georgiadis et al. (2018), a CCA spatial filter (Maye et al., 2017) and a beamformer as a spatial filter (Wittevrongel et al., 2017). Even though the results are still high, they suppressed the enriched frequency information of EEG activity, the brain rhythms. Every frequency can encode different cognitive functions related to a task, while the CFC between two frequencies can bind two different cognitive functions when it is demanded by the conditions of the experiment.

This work is the only one suggesting CFC features and namely PAC for both c-VEP (slow and fast) and SSVEP BCI systems. The proposed analytic scheme has been validated on three publicly available data sets with different designs and a different number of classes. Additionally, our results outperformed the ITR of the original data sets even by a factor of up to three (data set 3).

According to Klimesch's α theory, α ‘directs the information flow toward to neural substrates that encodes information related to the system’ (e.g., visual stimulus to visual system, voice/sound to auditory system). The physiological main function of α is linked to inhibition. Klimesch's α theory hypothesizes that α enables the storage of information via the inhibition of task-irrelevant neuronal substrates and by synchronizing the brain activity in task-relevant neural systems. Many research findings have shown that both evoked α and phase-locking further support the successful encoding of a global stimulus-based feature within the post-interval of 0–150 ms (Klimesch et al., 2011).

Apart from the cross low-frequency-high-frequency coupling (e.g., θ-γ), there is much evidence (Lakatos et al., 2005; Cohen, 2008; Isler et al., 2008; Voytek et al., 2010; Engel et al., 2013; Jirsa and Müller, 2013) that CFC can be observed between the lower frequency bands (e.g., delta-theta, delta-alpha and theta-alpha). Lakatos et al. (2005) made a hypothesis about the “hierarchy” of EEG oscillations, suggesting that the amplitude of a lower frequency band may be modulated by the phase of a higher frequency. They revealed, in the primary auditory cortex of macaque monkeys, that δ (1–4 Hz) phase modulates θ (4–10 Hz) amplitude, and θ modulates γ (30–50 Hz) (Lakatos et al., 2005). This multiplexity of brain rhythms might reflect a general trend in the organization of brain rhythms, a true evidence in both humans and cats (Bragin et al., 1994 widespread basis including the occipital brain areas in orienting acoustic responses where novel sounds intermixed with frequent standard and infrequent target (Isler et al., 2008).

Evidence from the human auditory cortex untangled that δ-band modulates the amplitude of θ-band ICMs, whose phase modulates the amplitude of γ-band ICMs (Schroeder et al., 2008). This indirect enhanced effect employs the spontaneous activity of local neural activity in the primary auditory cortex. Their hypothesis supports the notion that neural oscillations reflect rhythmic shifting of excitability states of neural substrates between high and low levels. This hypothesis is further supported by the fact that oscillations can be predicted by visual input such that the auditory input arrives during a high excitability phase and is finally amplified. In the present study, we demonstrated that the δ (0.5–4 Hz) phase modulates θ (4–8 Hz) amplitude over visual brain areas due to flickering images and their content and was mainly observed on parieto-occipital EEG recording sites.

We should also mention that the reason why δ^ϕ^ −θ^A^ coupling discriminates the flashing images can be directly linked to the content of the images. Visual attention sample image stimuli rhythmically demonstrate a peak of phase at 2 Hz (Dugué and VanRullen, 2014), while flashing images induce rhythmic fluctuations at higher frequencies (6–10 Hz) (Landau and Fries, 2012), here within the θ frequency range [4–8 Hz]. Finally, the work of Karakas et al. (2000) showed that the ERP represents an interplay between δ and θ frequencies and is directly linked to c-VEP (Demiralp et al., 2001).

δ-band oscillations long considered to be linked with deep sleep (Steriade, 2006). However, there are evidence that they play a key role in: (i) Controlling neuronal excitability, (ii) amplifying sensory inputs, (iii) in controlling and utilizing the attention, and (iv) unfolding the multiple operating modes responding to task demands (Schroeder and Lakatos, 2009).

Our results are aligned with findings in primary auditory cortex of macaque monkeys where δ (1–4 Hz) phase modulates θ (4–10 Hz) (Lakatos et al., 2005).

Ding et al. (2006) explored how attention modulates SSVEP power depending on the network triggered by the flickering frequency. They explored attentional effect at flicker frequencies within δ and α ranges. They found an occipital-frontal network to be phase-locked to the flicker when the flicker frequencies were within δ (2–4 Hz) and in upper α (10–11 Hz) when subject attending to the flicker. At flicker frequencies in the lower α (8–10 Hz), parietal and posterior frontal cortex, has higher amplitude when attention is directed away from the flicker. The major message from this study was that SSVEP amplitude and phase locking depends on which of two cortical networks, is selected by the flicker frequencies that have distinct spatial and dynamic properties.

There are strong evidence that slow-frequency ranges (δ, θ) play a pivotal role in controlling neuronal excitability and sensory processing and one would believe that play a key role also in attentional selection and especially during SSVEP (Morgan et al., 1996; Kim et al. (2007); Lakatos et al., 2008). There are findings that low-frequency oscillatory activity is enhanced by attentional demands during a task (Morgan et al., 1996; Kim et al. (2007); Lakatos et al., 2008). The coupling of δ-θ increased near visual stimulus onset during a visual attention task while it is decreased near visual stimulus onset in the auditory attention task (Lakatos et al., 2008). Finally, our results untangled that δ^ϕ^ −θ^A^ coupling over parieto-occipital brain areas is a valuable feature for the improvement of BCI performance and the related ITR.

## Conclusion

In this work, an efficient algorithmic approach was presented for two c-VEP-based BCI systems and a SSVE-BCI system with classes ranging from *N* = 6 to *N* = 32. We have demonstrated higher ITR in the three BCI systems outperforming the ITR presented in the original manuscripts. The proposed analytic scheme is based on CFC and namely PAC. Specifically, δ (0.5–4 Hz) phase modulates θ (4–8 Hz) amplitude and proved to be the candidate feature from PAC estimates that supported the highest classification accuracy, the fast ITR and the fast response time of the multi-class BCI systems.

Future improvements to the work presented could be the design of useful BCI-based application scenarios adapted to the needs of disabled subjects (King et al., 2014). Also, it might be useful to perform exploratory analysis on larger populations and in real time to further validate the results of the present work. Furthermore, many BCI systems based on c-VEP or SSVEP and tailored to different target populations could benefit from the current methodology to further improve ITRs (Lee et al., 2006).

## Ethics statement

The 3 datasets can be freely downloaded from the websites: Zurich (http://bci.epfl.ch/p300). Leuven: https://kuleuven.app.box.com/v/CVEP. Thessaloniki: https://physionet.org/physiobank/database/mssvepdb/.

## Author contributions

SD: Conception of the research; SD: Methods and design; SD and AM: Data analysis; SD: Drafting the manuscript; AM: Critical revision of the manuscript. Every author read and approved the final version of the manuscript.

### Conflict of interest statement

The authors declare that the research was conducted in the absence of any commercial or financial relationships that could be construed as a potential conflict of interest.

## Footnotes

^1^The data sets in Hoffmann et al. can be freely downloaded from the website of the EPFL BCI group (http://bci.epfl.ch/p300).

^2^https://kuleuven.app.box.com/v/CVEP

^3^https://physionet.org/physiobank/database/mssvepdb/
