# Supplementary material for: Enhancing Performance and Bit Rates in a Brain–Computer Interface System With Phase-to-Amplitude Cross-Frequency Coupling: Evidences From Traditional c-VEP, Fast c-VEP, and SSVEP Designs
Source: Front Neuroinform. 2018 May 8;12:19. doi: 10.3389/fninf.2018.00019 (PMC5952007; doi:10.3389/fninf.2018.00019)
Supplement: Supplementary file 1 [file Data_Sheet_1.DOCX]

Supplementary Material

Enhancing the Performance and Bitrates in Brain–Computer Interface System with Phase-to-Amplitude Cross-Frequency Coupling: Evidences from traditional c-VEP, fast c-VEP and SSVEP Designs

Stavros I.Dimitriadis^1-5*^, Avraam D. Marimpis^6^

^1^Division of Psychological Medicine and Clinical Neurosciences, School of Medicine, Cardiff University, Cardiff, United Kingdom

^2^Cardiff University Brain Research Imaging Centre, School of Psychology, Cardiff University, Cardiff, United Kingdom^3^School of Psychology, Cardiff University, Cardiff, United Kingdom

^4^Neuroinformatics Group, Cardiff University Brain Research Imaging Centre, School of Psychology, Cardiff University, Cardiff, United Kingdom

^5^Neuroscience and Mental Health Research Institute, Cardiff University, Cardiff, United Kingdom

^6^Brain Innovation B.V., Netherlands

1. **Comodulograms from the whole dataset of c-VEP**

**S1 - S3 illustrates the** trial-Averaged **PAC^iPLV^** patterns from the **c-VEP** responses for each target image and for both **attended vs non-attended images for the subjects 2 – 4 (disabled). S4 – S6 demonstrates the** trial-Averaged **PAC^iPLV^** patterns from the **c-VEP** responses for each target image and for both **attended vs non-attended images for the subjects 7 – 9 (able bodied).**

**
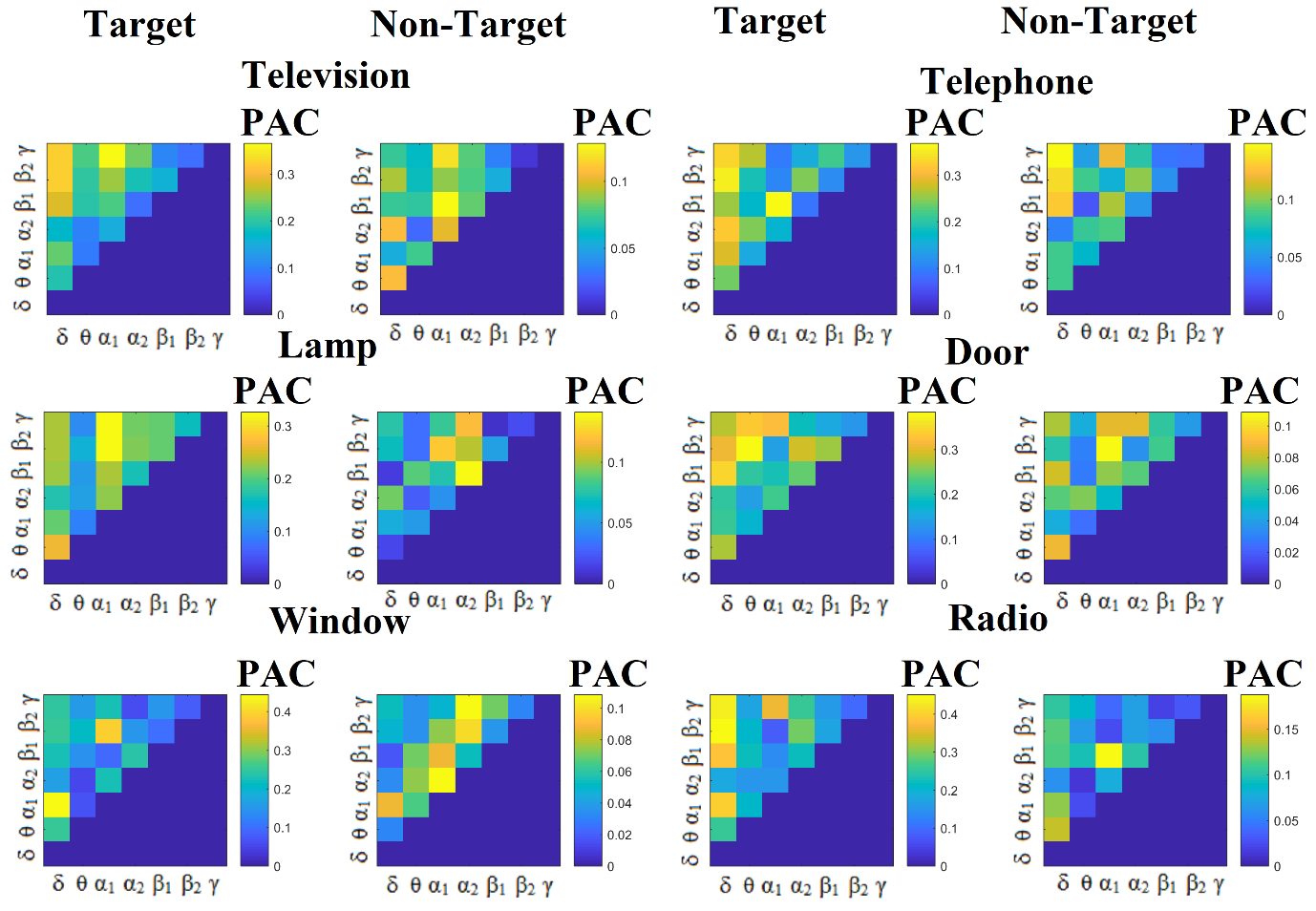
**

**S1. – subject 2 (disabled).**

**Demonstrating the level of CFC in c-VEP responses for each flashing image.**

Trial-Averaged **PAC^iPLV^** patterns from the **c-VEP** responses for each target image and for both **attended vs non-attended images.**

**
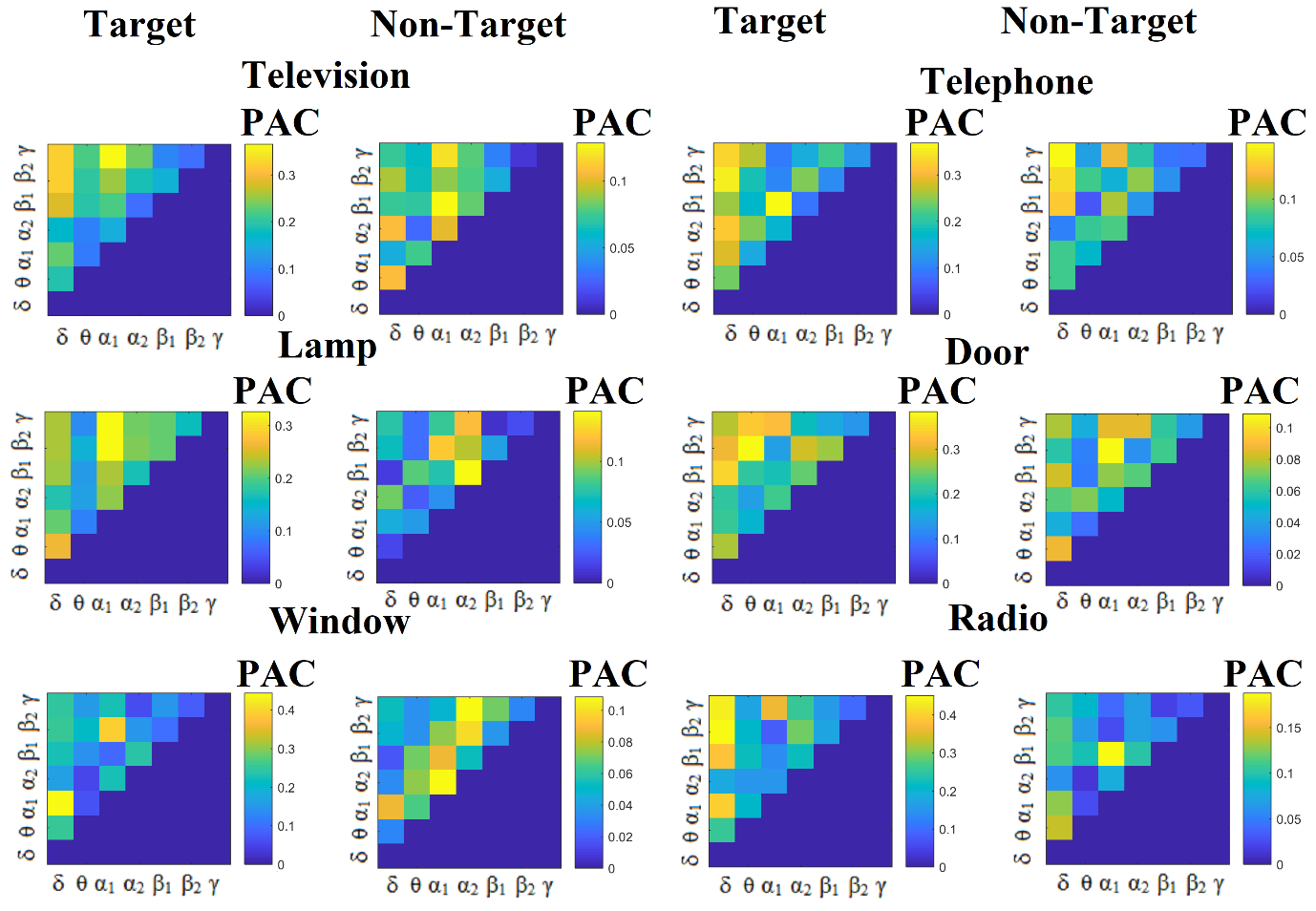
**

**S2. – subject 3 (disabled).**

**Demonstrating the level of CFC in c-VEP responses for each flashing image.**

Trial-Averaged **PAC^iPLV^** patterns from the **c-VEP** responses for each target image and for both **attended vs non-attended images.**

**
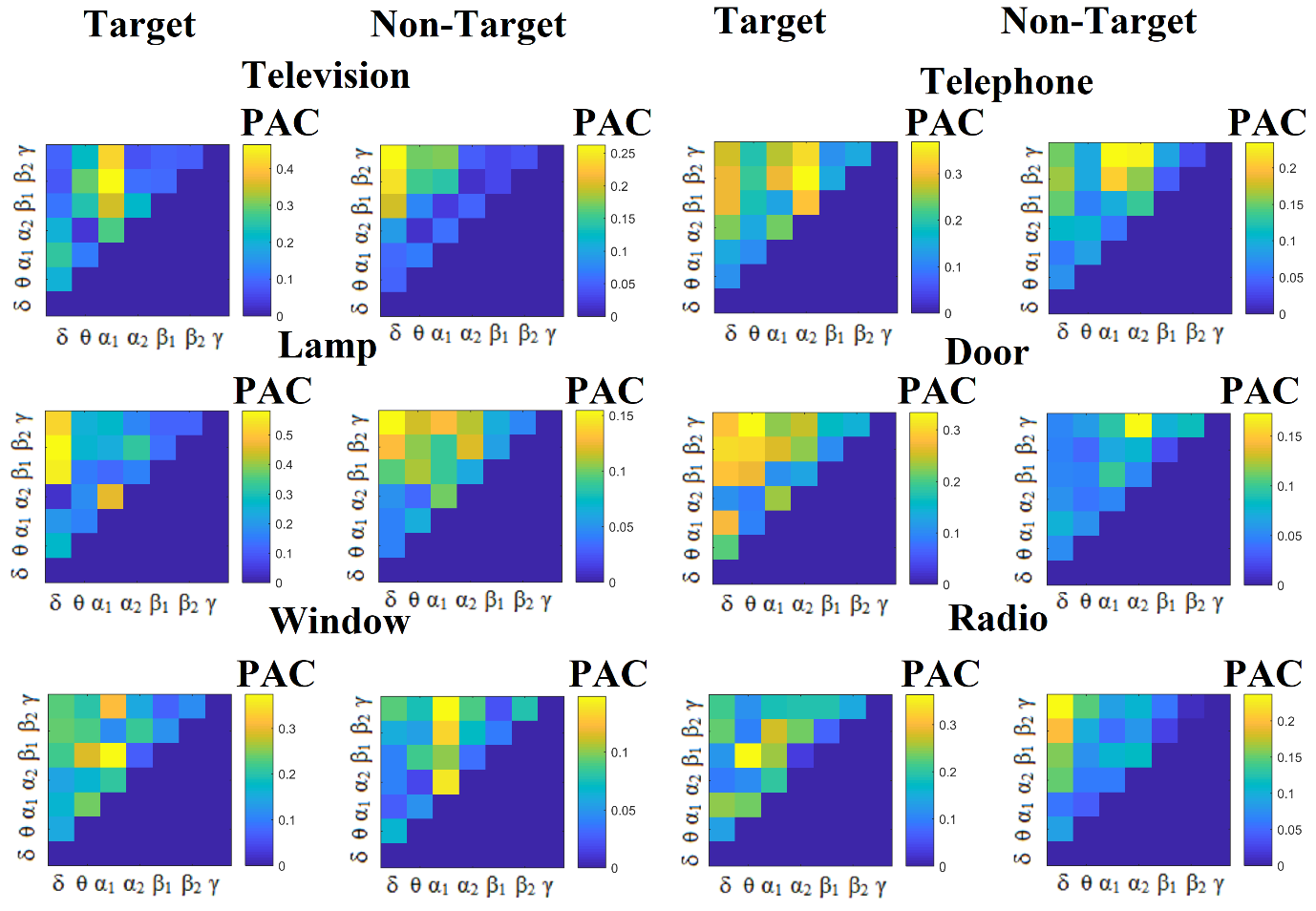
**

**S3. – subject 4 (disabled).**

**Demonstrating the level of CFC in c-VEP responses for each flashing image.**

Trial-Averaged **PAC^iPLV^** patterns from the **c-VEP** responses for each target image and for both **attended vs non-attended images.**


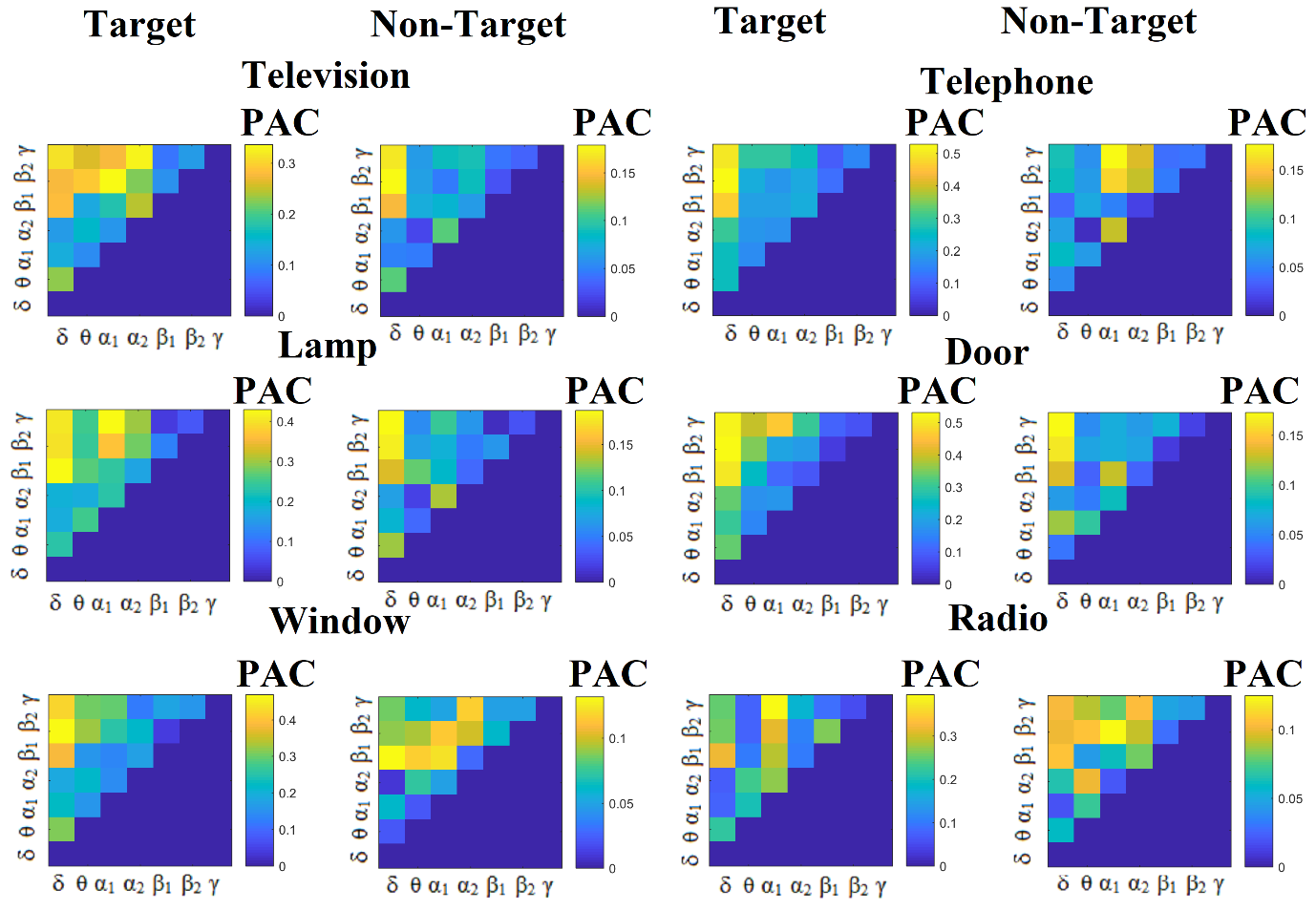


**S4. – subject 7 (able bodied).**

**Demonstrating the level of CFC in c-VEP responses for each flashing image.**

Trial-Averaged **PAC^iPLV^** patterns from the **c-VEP** responses for each target image and for both **attended vs non-attended images.**

**
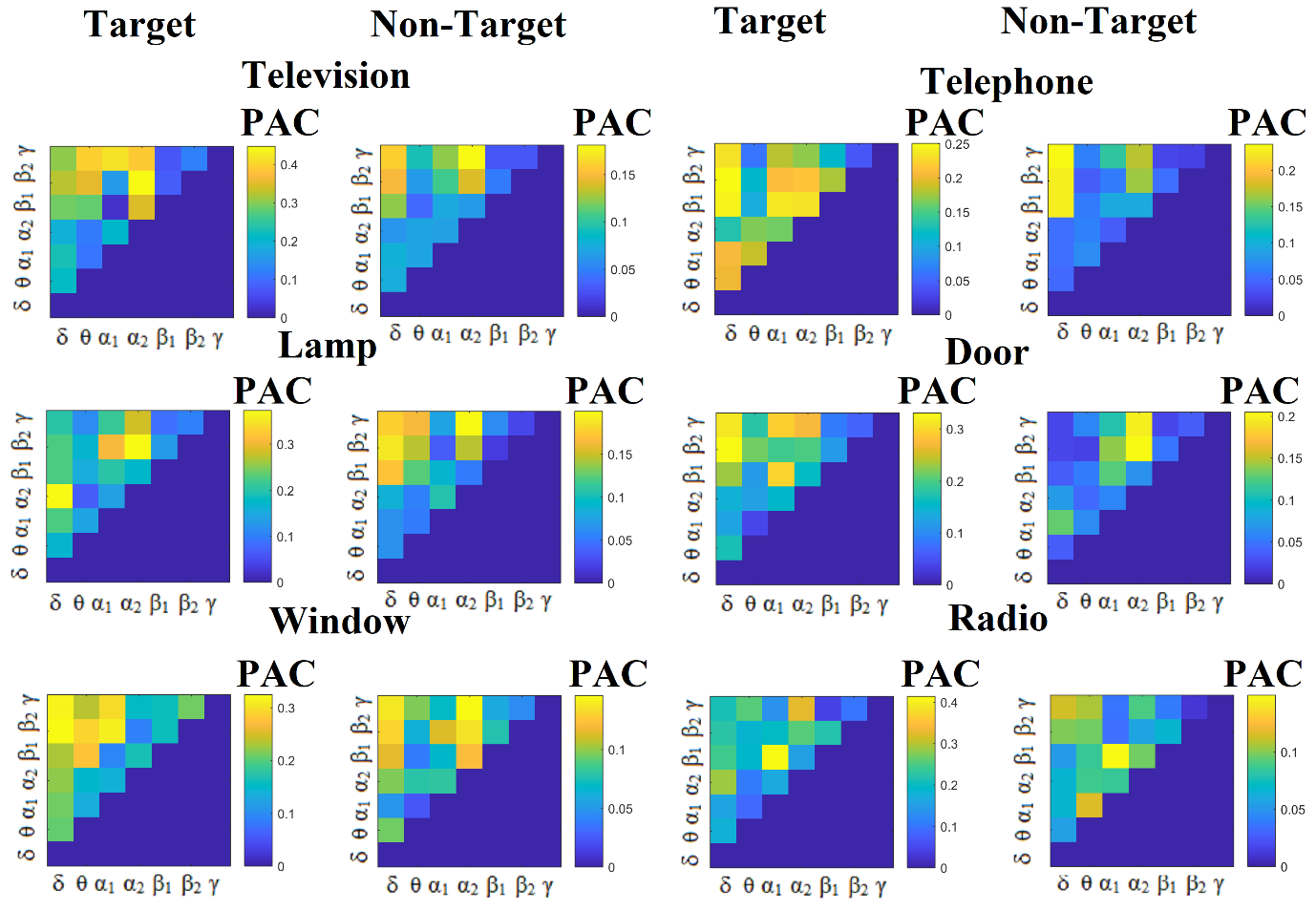
**

**S5. – subject 8 (able bodied).**

**Demonstrating the level of CFC in c-VEP responses for each flashing image.**

Trial-Averaged **PAC^iPLV^** patterns from the **c-VEP** responses for each target image and for both **attended vs non-attended images.**

**
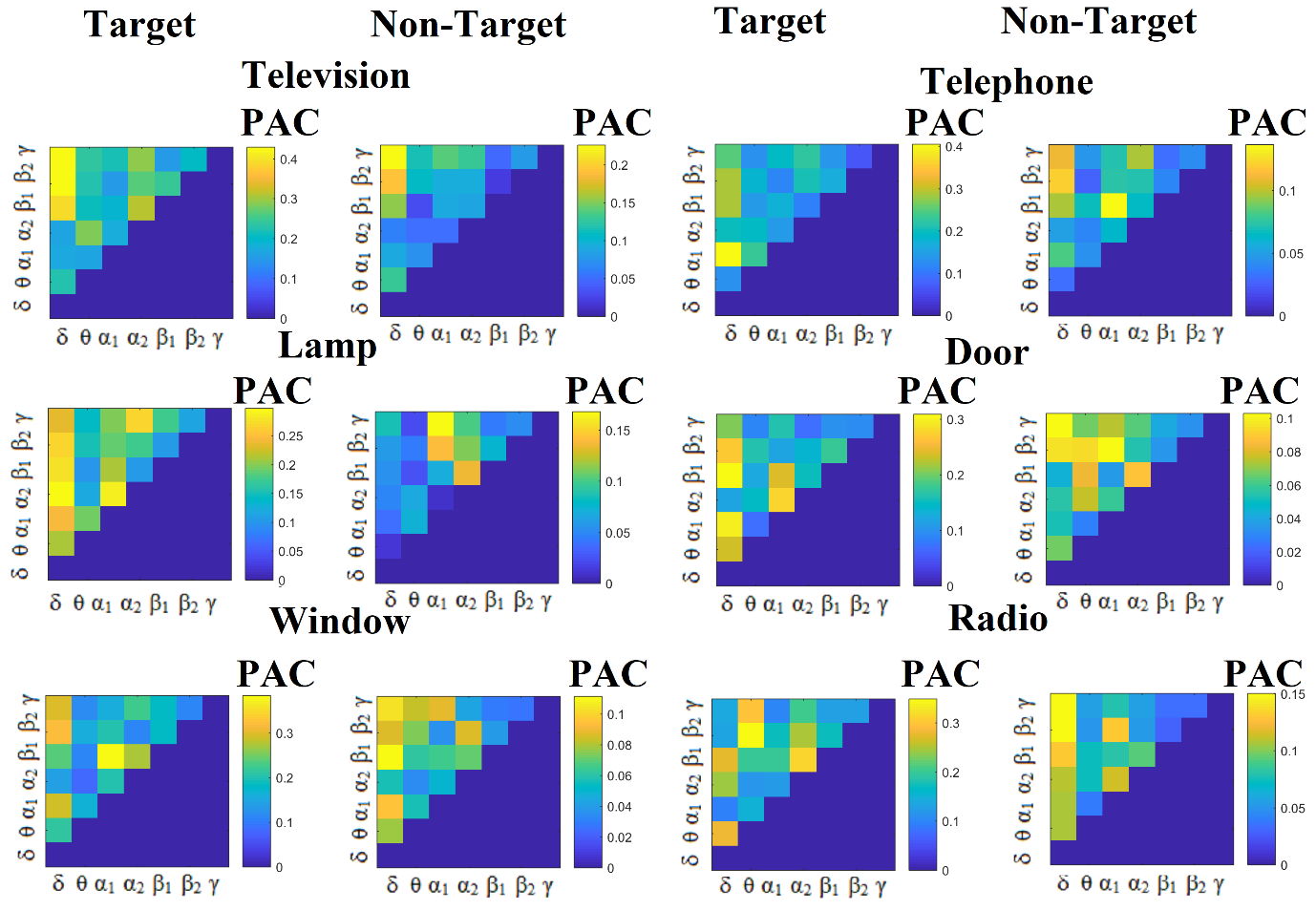
**

**S6. – subject 9 (able bodied).**

**Demonstrating the level of CFC in c-VEP responses for each flashing image.**

Trial-Averaged **PAC^iPLV^** patterns from the **c-VEP** responses for each target image and for both **attended vs non-attended images.**

1. **A Dissimilarity Measure for Dynamical Trajectories Based on the Wald-Wolfowitz (WW) Test**

The two-sample, non-parametric WW test was adopted in the present work to assess the degree of similarity between two nodal network metric time series based on global efficiency (nNMTS^GE^) derived from dynamic FCGs. The procedure entailed, first, transforming every pair of NMTS^GE^ time series *x(t), t* = 1.2,…T into dynamic trajectories represented by multidimensional vectors *X_t_* = [*x*(*t*), *x*(*t* + 1),…, *x*(*t* + *d_e_*)] and *Y_t_* = [*y*(*t*), *y*(*t* + 1),…, *y*(*t* + *d_e_*)] (*X* and *Y* correspond to two split-half segments from a single participant or from two participants). These vectors were formed by selecting the appropriate set of *d_e_*, which is the embedding dimension parameter that controls the dimensionality of the vectors and *d_t_* is the time-delay. By adopting the Ragwitz criterion, we optimized the embedding dimension *d_e_* and the embedding delay *d_t_* ([Ragwitz and Kantz, 2002](https://www.frontiersin.org/articles/10.3389/fninf.2017.00028/full#B87)), resulting in values ranging from 3 to 6 in both the complete and split-half temporal segments of NMTS^GE^ series. The two point-samples {X*_t_*}*_t_*_= 1:_*_m_* and {Y*_t_*}*_t_*_= 1:_*_n_* were then formed and the w_dist_ = w({X*_t_*},{Y*_t_*}) was computed.

Next, the minimal spanning tree (MST) graph of the overall sample was constructed (i.e., disregarding the sample identity of each point). In these graph points represent nodes with N − 1 edges (N = n + m) (i.e., paths within each pair of nodes). The second step of the procedure entails computing the R statistic which is the total number of consecutive sequences with identical sample identities (i.e., “runs”). Based on the number of edge pairs of MST sharing a common node and the degrees of the nodes, the mean and variance of R can be calculated ([Laskaris and Ioannides, 2001](https://www.frontiersin.org/articles/10.3389/fninf.2017.00028/full#B68)). This property of R permits computation of the initial form of the normally-distributed, WW Dissimilarity Index (w) as follows:

w=R−E[R]Var[R]√    (5)w=R-E[R]Var[R]    (1)

The measure used in classification schemes in the present work was derived from w using the Heaviside step function H(x) as follows: w_dist_ = |w|.H(−w). The higher the value of w_dist_, the more dissimilar the two point-sets are considered to be.

**References**

Laskaris, N. A., and Ioannides, A. A. (2001). Exploratory data analysis of evoked response single trials based on minimal spanning tree. *Clin. Neurophysiol.* 112, 698–712. doi: 10.1016/S1388-2457(00)00560-5

Ragwitz, M., and Kantz, H. (2002). Markov models from data by simple nonlinear time series predictors in delay embedding spaces. *Phys. Rev. E* 65:056201. doi: 10.1103/physreve.65.056201
